# Addressing uncertainty in modelling cumulative impacts within maritime spatial planning in the Adriatic and Ionian region

**DOI:** 10.1371/journal.pone.0180501

**Published:** 2017-07-10

**Authors:** Elena Gissi, Stefano Menegon, Alessandro Sarretta, Federica Appiotti, Denis Maragno, Andrea Vianello, Daniel Depellegrin, Chiara Venier, Andrea Barbanti

**Affiliations:** 1 Department of Design and Planning in Complex Environments, Università Iuav di Venezia, Venice, Italy; 2 Institute of Marine Sciences, National Research Council, Venice, Italy; University of Waikato, NEW ZEALAND

## Abstract

Maritime spatial planning (MSP) is envisaged as a tool to apply an ecosystem-based approach to the marine and coastal realms, aiming at ensuring that the collective pressure of human activities is kept within acceptable limits. Cumulative impacts (CI) assessment can support science-based MSP, in order to understand the existing and potential impacts of human uses on the marine environment. A CI assessment includes several sources of uncertainty that can hinder the correct interpretation of its results if not explicitly incorporated in the decision-making process. This study proposes a three-level methodology to perform a general uncertainty analysis integrated with the CI assessment for MSP, applied to the Adriatic and Ionian Region (AIR). We describe the nature and level of uncertainty with the help of expert judgement and elicitation to include all of the possible sources of uncertainty related to the CI model with assumptions and gaps related to the case-based MSP process in the AIR. Next, we use the results to tailor the global uncertainty analysis to spatially describe the uncertainty distribution and variations of the CI scores dependent on the CI model factors. The results show the variability of the uncertainty in the AIR, with only limited portions robustly identified as the most or the least impacted areas under multiple model factors hypothesis. The results are discussed for the level and type of reliable information and insights they provide to decision-making. The most significant uncertainty factors are identified to facilitate the adaptive MSP process and to establish research priorities to fill knowledge gaps for subsequent planning cycles. The method aims to depict the potential CI effects, as well as the extent and spatial variation of the data and scientific uncertainty; therefore, this method constitutes a suitable tool to inform the potential establishment of the precautionary principle in MSP.

## Introduction

Maritime spatial planning (MSP) is defined by the European Framework Directive on MSP 2014/89/EU as “a process by which the relevant Member State's authorities analyze and organize human activities in marine areas to achieve ecological, economic and social objectives" (art. 3 [1]) and considers the spatial allocation of maritime activities as the focus of the decision-making process [2].

Specifically, elaborating on marine spatial plans should be decided by analyzing “relevant existing and future activities and uses and their impacts on the environment, as well as to natural resources” (art. 4, comma 5). Objectives of MSP are declared in article 5, where the aim is to have sustainable development of maritime sectors (e.g., energy, transport, fishery, aquaculture, tourism and extraction of raw materials) coexisting with the preservation, protection and improvement of the marine environment, in coherence with the Marine Strategy Framework Directive 2008/56/EC (MSFD) [3]. The MSP Directive envisages the establishment and implementation of maritime spatial plans according to the ecosystem-based approach (EBA), in line with the MSFD provisions, and aims at “ensuring that the collective pressure of all activities is kept within levels compatible with the achievement of good environmental status” (art. 1(3)).

The MSP Directive considers applying the precautionary principle in Recital 14 when the following three preliminary conditions, as mentioned by the Commission on the precautionary principle (COM (2000) 1 final), are met: i) potentially adverse effects are identified; ii) the availability of scientific data is evaluated; and iii) the extent of scientific uncertainty is analyzed [4]. Cumulative impacts (CI) and uncertainty analyses can inform the application of the precautionary principle because they evaluate the effects of existing and potential human uses and pressures, data availability, and scientific uncertainty.

In 2014, the European Commission issued a macro-regional strategy for the Adriatic and Ionian Region (EUSAIR) (COM(2014)284 final) [5]. The aim is to support sustainable maritime economic development known as ‘blue growth’ in the region, focusing on activities such as aquaculture, fisheries, sustainable tourism, renewable energy sources, infrastructure and maritime transport. Under the EUSAIR framework, a pilot project called the ADRIPLAN (ADRiatic Ionian maritime spatial PLANning) was launched in 2014 to test an MSP process in the Adriatic and Ionian Region (AIR). The authors of the paper, as part of the planning team, implemented the CI assessment modelling in parallel to the planning process [6] to apply an ecosystem-based approach to MSP.

The CI assessment proposed by Halpern et al. [7] is the most widely used around the world [8]. Several approaches for mapping the cumulative impacts were analyzed by Judd et al. [9], with the aim of defining guidance for practitioners while implementing the CI assessments in their respective MSP pilots. Including the systematic analysis of limitations in the CI results is useful to define valuable information for implementing practical management measures [9,10], as the CI assessment holds great potential for science-based decision-making. A challenge of the CI assessment includes the “uncertainty in data and their combination" (p. 7) [11]. Several sources and causes of uncertainty are derived from data gaps and/or different data resolutions [11] and/or originated from incomplete knowledge and information [12]. Halpern and Fujita [11] concentrate on solutions to manage the data gaps in their work. However, the data gaps constitute only a portion of the uncertainty included in CI modeling, which includes all of the assumptions made in the modelling process [11,13].

As complex reality is imperfectly understood and reduced into models [14,15], uncertainty is inherent in any modelling approach, as in the application of the CI assessment in real situations [11,12]. Fundamentally, “uncertainties are communicated clearly, especially when integrating cumulative impact mapping into decision making, to ensure results are interpreted correctly” (p. 7) [9]. The effects of the CI model assumptions and the data quality models have been tested in isolation by previous studies [7,8,16,17]. Stock and Micheli [8] proposed a global uncertainty analysis to quantify uncertainty and depict what modelling factors (and related assumptions) mostly contribute to the uncertainty of the CI results. They demonstrated that model assumptions and data quality influence the results of CI assessment maps. Moreover, they noted that those influences depend on the case study region and the data describing it [8].

In this paper, we present the uncertainty analysis of a cumulative impact (CI) assessment for a pilot project in the AIR to inform the MSP effort. Similar to other studies, we compiled human use and environmental components for our analysis and calculated the CI scores based on sensitivity analyses from expert judgement. Additionally, as suggested by others [10,18], we propose a general uncertainty analysis structured in a three-level assessment to integrate problem identification, context framing and problem structuring (level 1 and 2) to subsequently tailor uncertainty (UA) and sensitivity (SA) analysis (level 3).

Uncertainty was initially assessed by applying and extending the approach of Walker et al. [13]. We describe the nature and the level of uncertainties by location and sub-location. We used expert judgement and elicitation to determine the sources of uncertainty related to the assumptions and gaps of the case-based MSP process in the AIR. Then, we used the results to tailor the global uncertainty analysis to spatially describe uncertainty distribution and variations. In this study, we assume that uncertainty is defined as “any departure from the unachievable ideal of complete determinism” [13].

The results of the CI scores and of the uncertainty analysis for the AIR are discussed for the level and type of reliable information and insights they provide to the MSP process. Moreover, we highlight a series of limitations and key issues that need to be considered if claiming adherence to the precautionary principle, as well as to reduce uncertainty in a possible subsequent planning cycle. The method can be adopted by decision makers to elaborate and negotiate with stakeholders about the thresholds identifying the acceptable risk of the potential CI. It also constitutes an operative method to guide the revision of available knowledge within the MSP adaptive process with new data and information on the greatest contributing uncertainty factors.

## Materials and methods

**Fig 1:**
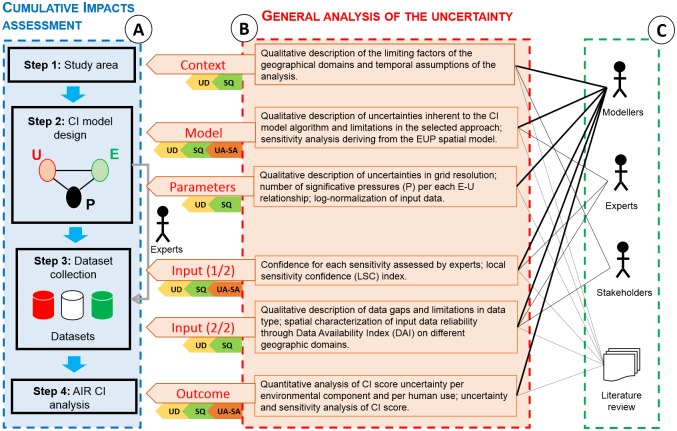
Analytical framework. Part A shows the Cumulative Impact assessment methodology; Part B refers to the general analysis of uncertainty based on the Walker et al. [13] uncertainty matrix and respective uncertainty quantification methods applied: UD = uncertainty description (level 1), SQ—Semi-Quantitative analysis (level 2); numerical uncertainty analysis (UA) and sensitivity analysis (SA) (level 3). In Part C, the contributors in the different modelling phases are reported.

This chapter is divided into two parts and presented in an analytical framework (Fig 1). Part A describes the methodology for the CI assessment in a case study for the AIR, where the following four steps are described: (1) Study area definition, (2) CI model design, (3) dataset collection and integration of expert knowledge for sensitivity score definition and (4) CI analysis on the AIR. Part B refers to the general analysis of uncertainty along the CI assessment starting from the application of the uncertainty matrix as suggested by Walker et al. [13]. For each uncertainty location, the respective uncertainty quantification methods, divided into three levels–(1) uncertainty description (UD), (2) semi-quantitative methods (SQ) and (3) numerical uncertainty methods (global uncertainty (UA) and sensitivity (SA) analysis (see section titled “General analysis of the uncertainty along the CI assessment process”)–are applied.

### Case study: Cumulative impacts assessment in the Adriatic-Ionian region

#### Step 1: Study area

In this step, the modelers defined the geographical domain and the temporal frame of the CI assessment of this study, which was derived from the EUSAIR domain of application within the ADRIPLAN MSP process.

**Fig 2:**
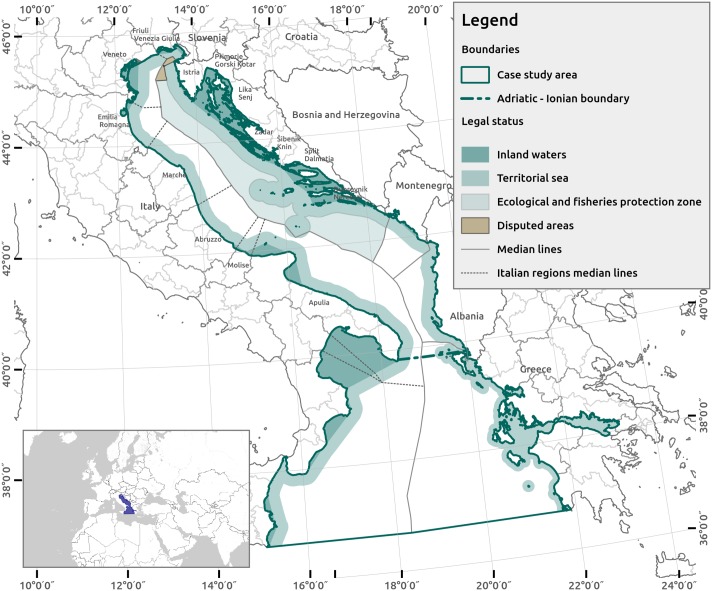
Case study area. The Adriatic and Ionian region.

The AIR is located in the eastern Mediterranean Sea and covers the entire Adriatic Sea (138,600 km^2^) and the northern portion of the Ionian Sea (199,000 km^2^) in the south (Fig 2). The Adriatic Sea is the largest shelf area of the Mediterranean [19]. The Adriatic and Ionian Sea communicate through the Otranto Strait, an approximately 72 km inlet that divides the Italian and Albanian coasts [20] has a maximum depth of 1,200 m [21].

The study area includes unique biological resources including *Posidonia oceanica* seagrass [22,23], coralligenous formations [24–26], nursery habitats for commercial species [27], and hosts marine vertebrates (dolphins, sea turtles, giant devil rays, whales and monk seals). From an administrative point of view, the study area is divided into seven riparian countries: four European Member States (Croatia, Greece, Italy, Slovenia) and three non-European countries (Albania, Bosnia-Herzegovina, Montenegro). The intensive anthropogenic activities scattered among seven countries with a high heterogeneity of geomorphological features and biological resources are a constant threat for biodiversity and the integrity of marine and coastal landscapes [28].

#### Step 2: Cumulative impact model design

In this study, we extended the cumulative impact (CI) model proposed by Halpern et al. [7] and later modified by Andersen et al. [29].

The case study area was divided into a regular square grid of 1 km^2^ (approximately 300,000 cells) using the EEA's reference grid for Europe [30,31] extracted for marine areas only. Andersen et al. [29] estimate the Cumulative Impacts index (CI) for a single grid cell as follows:

$$CI=\overset{l}{\underset{i=1}{\sum }}\overset{m}{\underset{j=1}{\sum }}\overset{n}{\underset{k=1}{\sum }}s(U_{i},\;P_{j},\;E_{k})\;i\left(U_{i},\;M\left(U_{i},\;P_{j},\;E_{k}\right)\right)d\left(E_{k}\right)$$

The CI model is based on human uses (U_i_), environmental components (E_k_) and pressures (P_j_). These sensitivity functions (U_i_, P_j_, E_k_) are the sensitivity of an environmental component E_k_ to a pressure P_j_ caused by an activity U_i_. The intensity function i(U,M) is the relative intensity of P caused by U in a grid cell according to spatial model M. Different from Halpern et al. [7], the pressures were derived by the MSFD (2008/56/EC) [4], considering pressures as factors causing temporary or permanent disturbances or damage to loss of one or several components of an ecosystem (S1 Table). The function M(U_i_, P_j_, E_k_) represents the spatial model for Pj caused by U_i_ on E_k_. For the spatial model (M), we modified the formula from Andersen et al. [29] as follows: whereas Andersen et al. applies M as a function of U and P (as M(U_i_, P_j_)), our model also depends on the environmental component as M(U_i_, P_j_, E_k_). Moreover, the spatial model M is based on a 2D Gaussian spatial convolution [32] instead of using a linear decay function as applied by others [12,29,33].

The probability function d(E) is the presence/absence of E_k_, which is 1 for a fixed E (seabed habitats) and varies from 0 to 1 for mobile special features (turtles, marine mammals and seabirds).

Different from the Andersen et al. [29] formulation, we introduced two additional factors: i) the response function *rf*, which represents the response of the ecosystem to stressors that can vary from a linear to a non-linear behavior [7,34–37]; and ii) the *mscf* factor, which was introduced for model dominant, additive and mitigative effects of multiple pressures in a grid cell. Although multiplicative effects have been studied in literature [38], we could not find any studies suggesting a multiplicative effects model that could be implemented for this study, as also considered by [8].

#### Step 3: Dataset collection

In the third step, input data were collected, with a total of 15 human activity datasets (S2 Table) and 31 environmental component datasets (S3 Table). All of the 46 spatial datasets were rasterized using the regular square cell grid of 1 km^2^ as described in Step 2.

The sensitivity scores were calculated through an expert survey using a structured questionnaire. In total, 99 regional experts from academia and research institutes were contacted by the planning team because of their proven knowledge on specific features included in the analysis. The experts were asked to evaluate sensitivities associating P from U to E through the following criteria: impact extent, impact level and recovery time, and buffer area (S4 Table). Moreover, for each sensitivity score, the experts were asked to express the confidence levels [c(U_i_, P_j_, E_k_)] as the level of reliability of their judgments based on empirical evidence, literature or personal knowledge and understanding [17,39,40].

#### Step 4: AIR cumulative impact analysis

In the fourth step, the CI scores were tested and mapped on the AIR assuming the following: i) the additive model for impacts are on the same grid cell (ci) [7]; ii) a linear response function; iii) the sensitivity scores, confidence and spatial model are derived directly from the expert judgement (described in step 3) [7,29,41]. This analysis, based on the assumptions from the literature, was used to inform the stakeholder and expert workshops to develop the general uncertainty analysis, as explained in the next section “General analysis of the uncertainty along the CI assessment process”. The CI and the subsequent analysis were performed using Tools4 MSP [42], an open source geospatial software package directly integrated in the ADRIPLAN Portal.

### General analysis of the uncertainty along the CI assessment process

**Fig 3:**
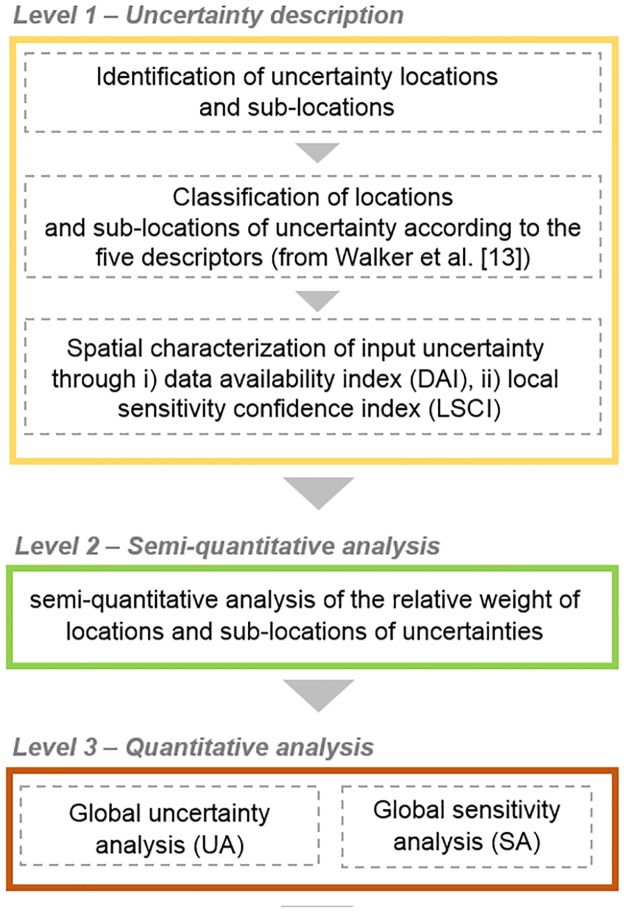
Scheme of the three-level analysis of uncertainty. Modelers initially describe uncertainty in level 1. Semi-quantitative analysis is performed in level 2, while statistical quantitative analysis in level 3.

We developed a three-level analysis of uncertainty to address limitations and model assumptions for the AIR case study area and to identify the information needs for the MSP process in the AIR (Fig 3). Level 1 identifies uncertainty in general terms on the entire AIR and along the CI assessment process. Level 1 considers the description of (i) uncertainty locations and sub-locations; (ii) sources of uncertainties per location according to 5 descriptors defined by Walker et al. [13] and (iii) the spatial characterization of the inputs’ uncertainty (section “Level 1: Locations and the level and nature of uncertainty with spatial characterization”).

Level 2 applies a semi-quantitative analysis of uncertainty (SQ) through expert elicitation to rank model locations and sub-locations based on the uncertainty magnitude (section “Level 2: Semi-quantitative analysis of uncertainty for the CI assessment”). The analysis is applied to the entire AIR and for the entire CI assessment.

Level 3 performs the global uncertainty (UA) and sensitivity (SA) analysis, considering the potential effects from the simultaneous variation of parameters related to the selected locations sub-set. While UA is performed on the entire AIR, SA is limited to the sub-areas of the AIR, and on a sub-set of locations emerging from level 1 of analysis, for which numerically quantifying the uncertainty is possible (section “Level 3: Uncertainty analysis and sensitivity analysis”).

#### Level 1: Locations and the level and nature of uncertainty with spatial characterization

The uncertainty analysis for level 1 applied the uncertainty matrix proposed by Walker et al. [13,22] for the CI assessment. The matrix provides a systematic approach to identify and classify uncertainties specific to a particular modelling process, as applied to a study region and related dataset. In Table 1, the five locations of uncertainty (context, model, input, parameters and outcomes) are presented, which describe the phases or decision nodes of the modelling activities where uncertainty manifests itself [13]. Each location is qualified by five descriptors (Table 2) and grouped by level (statistical uncertainty, scenario uncertainty and recognized ignorance) and by nature (epistemic and variability nature).

**Table 1:** Locations of uncertainty.

Locations of uncertainty	General definitions
**Context**	conditions and circumstances (and even the stakeholder values and interests) that underlie the choice of the boundaries of the system, and the framing of the issues and formulation of the problems to be addressed within the confines of those boundaries
**Model**	model structure uncertainty: lack of sufficient understanding of the system (past, present, or future) in current behavior or future evolution, entailing issues related to system boundary, functional forms, definitions of variables and parameters, equations, assumptions and mathematical algorithms model technical uncertainty: generated by software or hardware errors
**Inputs**	data that describe the reference (base case) system the external driving forces that have an influence on the system and its performance
**Parameters**	(exact, fixed, a priori chosen, calibrated)
**Outcome**	Results of the modelling process

Locations of uncertainty represent where the uncertainty manifests itself within the model complex; adapted from Walker et al. [13].

**Table 2:** Descriptors of uncertainty.

Descriptors of uncertainty	Definitions
**1. Level**	where the uncertainty manifests itself along the spectrum between deterministic knowledge and total ignorance
**1.1 Statistical uncertainty**	any uncertainty that can be described adequately in statistical terms, as sampling error, or inaccuracy or imprecision
**1.2 Scenario uncertainty**	related to the process of making assumptions that in most cases are not verifiable, so associated with uncertainty at a level beyond statistical uncertainty; it entails a range of possible outcomes, but the mechanisms leading to these outcomes are not well understood and it is, therefore, not possible to formulate the probability of any one particular outcome occurring
**1.3 Recognized ignorance**	fundamental uncertainty about the mechanisms and functional relationships being studied
**2. Nature**	Whether the uncertainty is due to the imperfection of our knowledge or is due to the inherent variability of the phenomena being described.
**2.1 Epistemic uncertainty**	The uncertainty due to the imperfection of our knowledge, which may be reduced by more research and empirical efforts.
**2.2 Variability uncertainty**	The uncertainty due to inherent variability, which is especially applicable in human and natural systems and concerning social, economic, and technological developments.

Descriptors of uncertainty qualify the level and nature of uncertainty per each location; adapted from Walker et al. [13].

Initially, the modellers divided the locations of uncertainty into sub-locations, depending on the assumptions, hypothesis and decision nodes made while building the CI assessment model. In total, 17 sub-locations were defined (S5 Table), reflecting the potential sources of uncertainty. In addition, the uncertainty matrix was discussed in the first core expert workshop (Venice 12/05/2014) with the ADRIPLAN Project partners and was composed by a panel of interdisciplinary experts in ecology and environmental sciences and planners and biologists who provided the input data for the analysis. The aim was to consolidate the uncertainty matrix by identifying gaps within a wider expert group.

During the uncertainty elicitation process, the following four methods were applied: i) a literature review, ii) direct interviews with 11 experts concerning environmental input data (seabed habitats, marine mammals, seabirds, sea turtles and giant devil rays), iii) surveys that included 99 experts and iv) six stakeholder meetings (150 participants [6], from which 40 regional experts met in various locations Venice on 29/09/2014 and 10/07/2015, Corfu on 27/06/2014, Trieste on 07/07/2014, Pola on 04/03/2015, and Lecce on 11/03/2015). During the stakeholder meetings, the initial results and finding on the CI assessment from the baseline run and uncertainty analysis were presented during workshop sessions. The participants were asked to comment on gaps and limitations on both analyses during the sessions or through personal communications with the modelers along with the meetings. Feedback on the locations and on the description of uncertainties were collected using the four methods and organized by the authors in the CI assessment uncertainty matrix, tracked and reported according to the five uncertainty descriptors.

Once the CI assessment uncertainty matrix was consolidated, we extended the approach of Walker et al. [13] by introducing the following two spatial-explicit descriptive indicators of the input uncertainty sub-locations: i) the data availability index (DAI) and ii) a local sensitivity confidence index (LSCI).

The DAI defines the spatial distribution of the available input datasets (S2 and S3 Tables), inherent to their geographical coverage of the selected environmental components and human use dataset. Input information for the DAI was a gazetteer of 22 terms describing the type and geographical realms of each dataset (S1 File). The 22 terms were organized according to a scale in the marine regions (Adriatic, Ionian), the national (per country) and sub-national domains (only for Italy and in administrative Regions), which represent areas under a specific country’s jurisdiction or portions of a specific country’s jurisdiction. The DAI is calculated as the average sum of terms related to the spatial distribution of data availability as follows:

$$DAI=\frac{\sum_{i=1}^{l}a\left(U_{i}\right)+\sum_{k=1}^{n}a\left(E_{k}\right)}{l+n}$$

where the function a(U_i_) is the availability of spatial information of the use U_i_, and the function a(E_k_) is the availability of spatial information of the environmental component (E_k_).

The LSCI defines the spatial reliability of the CI score per grid cell in relation to the knowledge level expressed by experts on the sensitivities through the confidence of each E-U-P relationship. The LSCI is calculated as the weighted average on cumulative impact score ci(U_i_, P_j_, E_k_) on each grid cell as follows:

$$LSCI=\frac{\sum_{i=1}^{l}\sum_{j=1}^{m}\sum_{k=1}^{n}c\left(U_{i},\;P_{j},\;E_{k}\right)*ci\;\left(U_{i},\;P_{j},\;E_{k}\right)}{\sum_{i=1}^{l}\sum_{j=1}^{m}\sum_{k=1}^{n}ci\;\left(U_{i},\;P_{j},\;E_{k}\right)}$$

where c(U_i_, P_j_, E_k_) represents the confidence in experts’ judgement on sensitivity. The LSCI represents the distribution of areas where our sensitivities knowledge and understanding is higher, according to the state of the art synthesized by the expert survey. From the LSCI analysis, we ranked the sensitivities scores with respect to their contribution in the CI model output.

#### Level 2: Semi-quantitative analysis of uncertainty for the CI assessment

**Fig 4:**
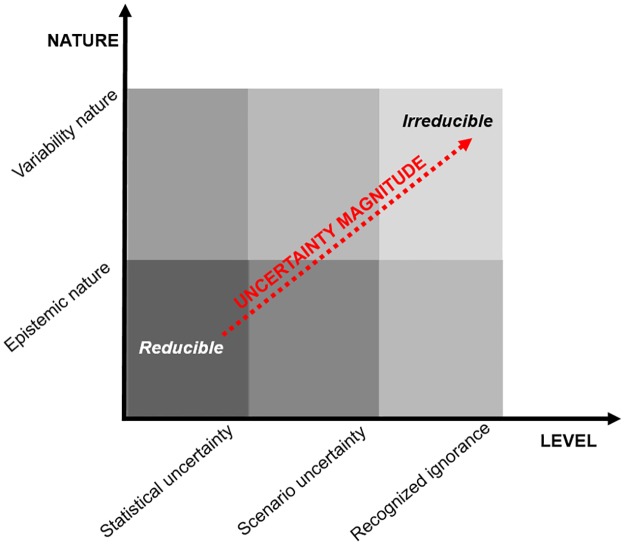
Uncertainty magnitude. Combinations between level and nature of uncertainty give place to 6 different types of uncertainty magnitudes; elaborated from [10,13].

Semi-quantitative (SQ) analysis is a technique used to generically assess and rank the weight of uncertainty descriptors (level and nature) per sub-location on models. The SQ methodology was proposed and used by Stelzenmüller et al. [10] to rank locations of uncertainty in a set of models for monitoring and assessing marine spatial management plans. We combined level and nature to generate 6 different types of uncertainty (Fig 4). Uncertainty magnitude ranges from epistemic nature, which is reducible, to variability nature, which is irreducible because it is inherent to system variability [13].

Initially, per sub-location of the CI uncertainty matrix, a score of 1 (presence) or 0 (absence) was associated per descriptor, as modified from Stelzenmüller et al. [10]. When multiple sources of uncertainty per sub-location were identified, multiples of 1 (presence) were associated for the specific sub-location and descriptor. Next, the uncertainty descriptors were scored between 1 and 3 per their level and nature, according to the uncertainty magnitude they represent [10,13].

The final score of uncertainty per sub-location was obtained from the sum of the different sources’ scores of uncertainty multiplied by the factors for each descriptor. The results were visualized to describe and synthesize in semi-quantitative terms the relative weight of the different sub-locations of uncertainties in the CI assessment by considering the following three factors: i) the distribution of the uncertainty rate in percentage per the location and sub-location, ii) the relative weight of the five uncertainty descriptors for the CI assessment, and iii) the relative weight of uncertainty per sub-location according to the 5 uncertainty descriptors.

#### Level 3: Uncertainty analysis and sensitivity analysis

In level 3, we performed a global uncertainty (UA) and sensitivity (SA) analysis to quantify the simultaneous effects of the variation of factors identified in level 1. The UA describes the entire set of possible CI scores of the CI assessment model together with their associated occurrence probability. The SA determines the change in the model’s output values as a function of the model input values. The UA was applied to the entire AIR, while the SA was only applied to the geographical areas where there were no significant data gaps that emerged from the level 1 analysis results and DAI (Fig 3).

In accordance with Stock and Micheli [8], we used global methods that assess the effects of all factors simultaneously, including their interactions. Different from Stock and Micheli [8], we used the “Sobol’ indices” [43] that express the share of variance of the output model that is due to a given input or input combination. Additionally, we also used the 'total effect’ index that provides a measure of the total effect of a given factor, including all of the possible synergistic terms between that factor and all the other factors [44]. Table 3 summarizes the four factor groups, the factors included in the analysis and their respective range in the Monte Carlo (MC) simulation. The adopted CI model contains a high number of input factors (e.g. a sensitivity score and a model distance for each U, P, and E combination). This was required for variance-based measures such as Sobol’ indices and can be flexibly adjusted to work with groups of factors, for example, to produce an overall sensitivity measure relative to a group [45].

**Table 3:** Factor groups, factors and factor ranges applied in the Monte Carlo (MC) simulations in this study.

Factor group	Factor	Range in MC simulations
**SC**^a^	s(Ui; Pj; Ek): sensitivity score errors	Errors from beta distribution B(s, c) with s sensitivity score (modal value) and confidence (variance) from expert judgement ranging from 0 to 1.
**D**^a^	M(Ui; Pj; Ek): pressure distance errors	Errors from beta distribution B(d, c) with d pressure distance (modal value) and confidence (variance) from expert judgement ranging from 0 to 50km.
**MSCF**	MSCF: multi-stressor combination factor	Values from uniform distribution U(0, 1) where multi-stressor combination varies from MSCF = 0 for additive model, to MSCF = 1 for dominant model.
**RF**^b^	NR: nonlinear response factor	Values from uniform distribution U(0, 1) where the response of ecosystem components varies from NR = 0 (linear response) to NR = 1 (threshold response).
	SR: skewness response factor	Values from uniform distribution U(0.3, 0.7) [8]. For low SR values the impact occurs at low levels of pressure; for high SR values the impact occurs at high levels of pressure.

^a^ 60 most weighted U_i_P_j_E_k_ relationship according to the CI baseline run results.

^b^ Response factor.

To estimate Sobol’ indices, a quasi-Monte Carlo sampling strategy was adopted. The quasi-random sampling method reduces the number of simulations required to attain a given accuracy in the sensitivity estimates [46]. For first order (no interaction between factors), second order (interaction between two factors) and total indices (interactions among all factors), we applied Saltelli’s [47] methodology. This method yields a more robust sensitivity estimation than other methods such as analysis of variance or regional sensitivity analysis [48–50]. To obtain a spatial representation of the input factors’ uncertainty, the Sobol’ indices analysis was performed for each grid cell. This allows identification of the local variation as influenced by each factor of the model’s uncertainty [8,50,51].

The UA and SA follow a common workflow that can be summarized in the following six phases [50]: (1) Defining the target function of the UA and SA as the CI score rescaled to a 0–1 range per grid cell to evaluate the relative variation of the CI of the run simulations; (2) Selecting the input factors to be analyzed; (3) Assigning a statistical distribution to the selected input factors (see Table 3), taking into account the level of confidence expressed by experts introducing a more suitable probability distribution of the single factor. For factors’ sensitivity score errors (SC) and pressure distance errors (D), we adopted the beta-distribution (B(s, c)) assuming the modal values from expert judgment on sensitivities (s) and the variance from the confidence (c) (S1 Fig); (4) Applying a sampling design for the quasi-Monte Carlo simulation; (5) Performing 15,000 simulation runs; and (6) Determining the UA and SA of the simulation results by assessing two factors: (i) the spatial representation of the UA through the coefficient of variation, and for each grid cell, the greatest (25%) and least (10%) impacted which were retained and mapped [8,52]; and (ii) the first-order, second-order and total effects sensitivity measures for each grid cell which were estimated and analyzed.

## Results

### Cumulative impact score for the AIR

**Fig 5:**
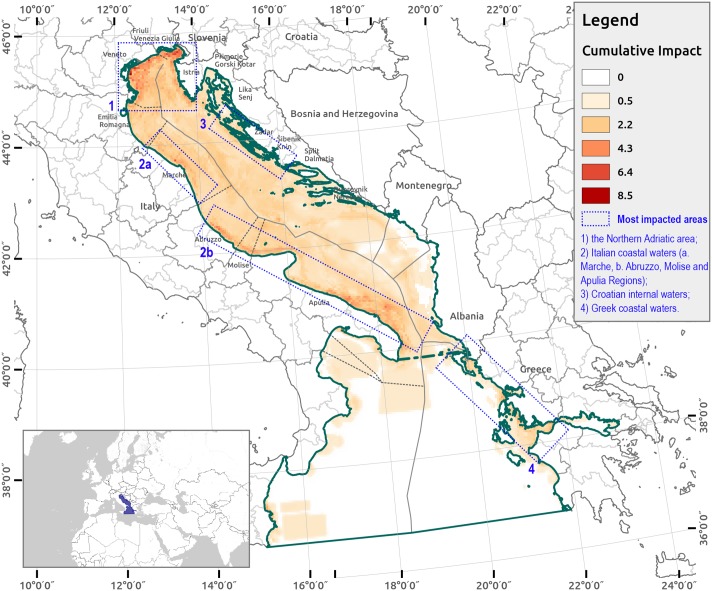
Cumulative impacts scores derived from the baseline run for the AIR. Cumulative impact scores varies from 0.0 (no impact) to 8.5; most impacted areas are indicated in blue frames.

The baseline run of the CI model (Fig 5) identifies the following four areas of high anthropogenic impact: 1) the Northern Adriatic area; 2) Italian coastal waters (Marche, Abruzzo, Molise and Apulia Regions); 3) Croatian internal waters; and 4) Greek coastal waters.

**Fig 6:**
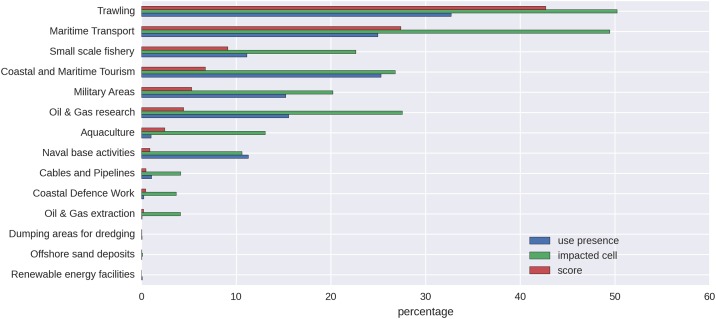
Contribution of human uses (U) to the CI scores for the AIR. “Use presence” represents the percentage of the AIR where the use is located; “impacted cells” represents the percentage (%) of cells that are impacted by the use, considering the distance model at which the pressure takes place; “scores” represents the contribution to the total CI score of the use in percentage.

In Fig 6, the human uses (U) with the highest contribution to the CI score are presented. Trawling covers 33% of the cells, and it impacts approximately 50% of the AIR because of the distance models at which pressures take place, contributing 43% of the total CI score. Maritime transport covers approximately 25% of the cells, and it impacts almost 50% of the AIR, contributing 28% of the total CI score.

**Fig 7:**
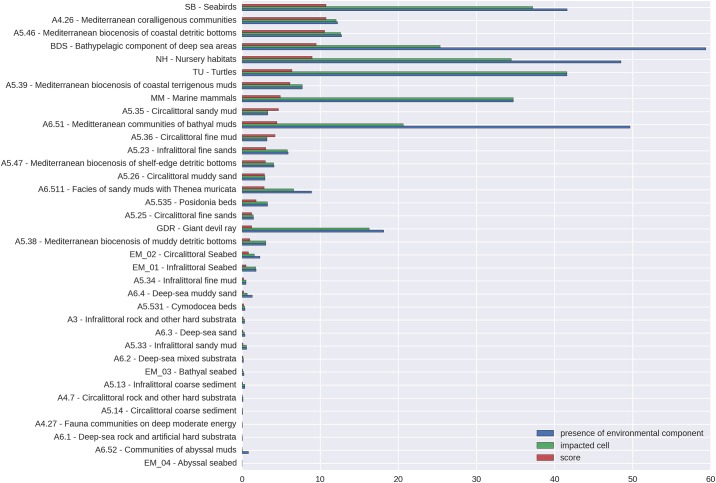
Ranking of the environmental components (E) that are majorly affected by the CI scores in the AIR. “Presence of environmental components” represent the percentage of the total cells where E is located, “impacted cells” represents the percentage of cells where E is located that are impacted by U, “score” represents the contribution to the total CI score deriving from E.

In Fig 7, the impact on the environmental components (E) is presented. Seabirds, Mediterranean coralligenous communities (A4.26), and Mediterranean biocenosis of coastal detritic bottoms (A5.46) were most affected by human pressures in the AIR, each contributing 11% to the cumulative impact score.. The modelling results show that the following 12 E are fully impacted across their entire spatial coverage in the AIR: Mediterranean coralligenous communities (A4.26), Mediterranean biocenosis of coastal detritic bottoms (A5.46), sea turtles, Mediterranean biocenosis of coastal terrigenous muds (A5.39), marine mammals, circalittoral sandy muds (A5.35), circalittoral fine muds (A5.36), infralittoral fine sand (A5.23), circalittoral muddy sand (A5.26), *Posidonia* beds (A5.535), circalittoral fine sands (A5.25), and Mediterranean biocenosis of muddy detritic bottoms (A5.38).

### General analysis of the uncertainty along the CI assessment process

#### Locations and the level and nature of uncertainty with spatial characterization (level 1)

The CI uncertainty matrix with a detailed description of the level and nature descriptors per sub-location is reported in S6 Table. For the 17 sub-locations of uncertainty, 31 sources of uncertainties differing in level and nature were detected and described.

With respect to the context location, the main scenario uncertainties are primarily related to the geographical domain of the EUSAIR as a spatial domain of the MSP process and in relation to the consistent implementation of the CI model. Moreover, the analysis does not include any reference to the seasonal or even the monthly variability of the CI mechanisms mostly related to the special features and their seasonal dynamics (e.g., spawning areas). In contrast, the values are recorded statically for the reference year (2016).

Uncertainties in the model location include the following 4 factors: i) the lack of proper oceanographic models representing the impacts dispersion mechanisms in the Adriatic and Ionian seas, both according to the surface circulation [53], the deep circulation between the Adriatic and Ionian [54] and with the bordering marine areas [55]; ii) the representativeness of the spatial models of pressures on E, iii) the fact that the baseline environmental conditions are not considered; and iv) the representativeness of the response of E to P, which is considered homogeneous on each E everywhere (as mentioned previously by Korpinen et al. [41]). Recognized ignorance is reported for the response of E to P, where the variability in the level of resilience and identification of regime shifts is mentioned in the literature [56–58].

Uncertainties related to model inputs are reported for U, E and E-U-P sensitivities as follows: i) missing datasets for some U and for land-based pollution; ii) for E, limited dataset coverage in the Ionian for marine mammals and giant devil rays, dataset proxies for marine mammals and turtles, and sensitivity analysis on the EMODnet dataset for seabed habitats [59]; and iii) for the E-U-P sensitivities, the ecological meaning and the method of sensitivities assessment including the recognized ignorance for the E-U-P relationship for some E, for example, the deep sea and seabirds.

The parameters’ uncertainties are related to i) the grid resolution with respect to the dataset resolution, ii) the number of significant pressures per E-U relationship, and iii) the log-normalization of certain datasets.

Uncertainties of the CI outcomes derive from the combination and dispersion of statistical uncertainties of the input data but also from the spatial models emerging from the combination of each E-U-P relationship. Moreover, the recognized ignorance is related to the mechanisms of multiple stressors in combination (e.g., synergistic and multiplicative or reducing effects), as demonstrated by Halpern et al. [7]) and to the ecological significance of the potential impacts on E, considering not only the presence of high CI scores, but the effects of low CI signals at the population or community level for vast areas throughout the long term as emerging from the CI results.

**Fig 8:**
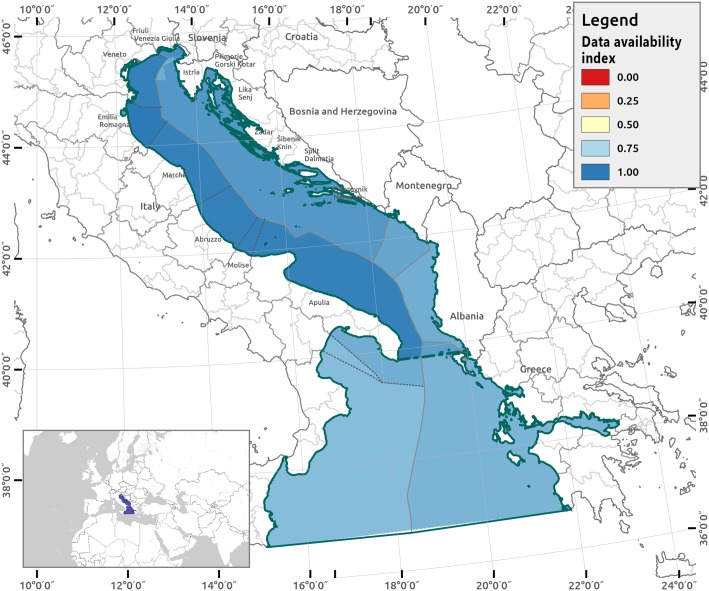
Data availability index (DAI) for the AIR. Dark blue indicates where all data sets are available.

In Fig 8, results from the Data Availability Index (DAI) are reported. The marine area of the Italian Adriatic is mostly covered by all of the datasets (DAI = 14).

Dataset distribution varies according to the geographical areas as reported in S7 Table. With respect to E, the Adriatic sea is covered by all 36 datasets, while for the Ionian Sea, the following 2 datasets are missing: i) marine mammals, and ii) giant devil rays (panel A in S2 Fig). With respect to U, the geographical domains covered the least include Slovenia and Albania in the Adriatic and the Ionian Sea in general. Only 5 U datasets cover the entire AIR (cables and pipelines, LNGs, renewable energy facilities, naval based activities, and trawling) (panel B in S2 Fig). The least represented U in the model relates to dumping areas for dredging, which is available only for the Emilia Romagna Region in the Northern Adriatic.

**Fig 9:**
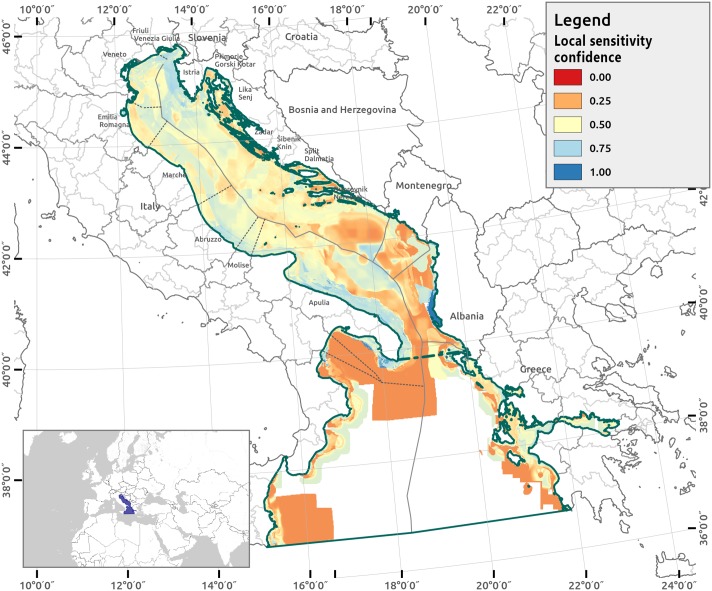
Local sensitivity confidence index (LSCI) for the AIR. LSCI = 1.00 (in dark blue) indicates where the LSCI is higher, meaning that the confidence in sensitivities judgement from experts is high; LSCI = 0.00 (in dark red) indicates where the LSCI is lower, meaning that the confidence in sensitivities judgement from experts is low.

Fig 9 reports the Local Sensitivity Confidence Index (LSCI) distribution in the study area. High confidence areas (≥ 0.8) cover 1.20% of the impacted cells. These areas are located in the territorial waters, mainly in front of the Apulia Region, Albania and Greece. The sensitivity scores that contribute 90% of the total CI are reported in S4 Fig.

#### Semi-quantitative analysis of uncertainty (level 2)

**Fig 10:**
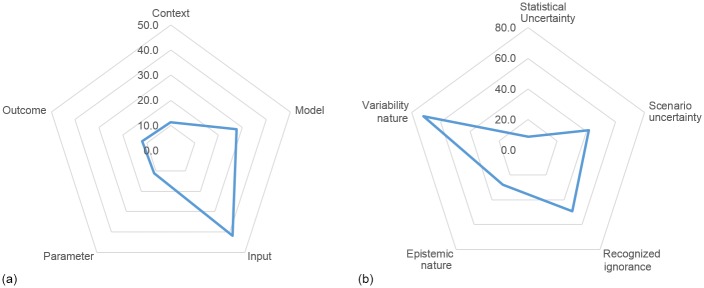
Distribution of the rate of uncertainty in percentage per location (a) and per level and nature (b).

Considering the 31 sources of uncertainty distributed in the 17 sub-locations, the CI assessment model is affected by uncertainty mainly in the model input (41.9%) and in the model algorithm (27.5%), while the uncertainty of context, parameter and output counts for approximately 11.5% (Fig 10A). Uncertainty primarily is a variable of nature (72%) rather than epistemic nature (28%) (Fig 10B). Uncertainties can be reported under a recognized ignorance (49.3%) and as a variable of nature (41.8%). Statistical uncertainty accounts for only 9.0% (Fig 10B).

**Fig 11:**
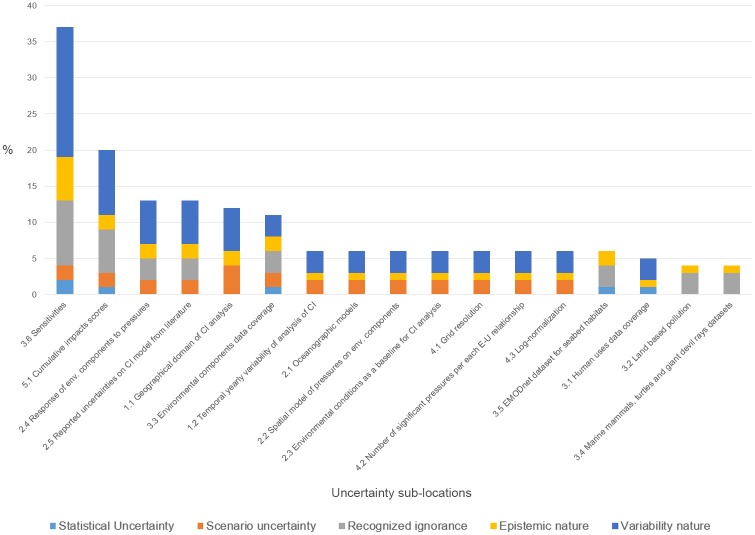
Relative weight of uncertainty per sub-location according to the 5 uncertainty descriptors. Ranking of sub-locations according to the relative weight of uncertainty.

The relative rank of uncertainty distribution for the 17 sub-locations is illustrated in Fig 11. The highest amount of uncertainty is due to the E-U-P sensitivities, characterized by uncertainty of variability nature and recognized ignorance. The next three major sources of uncertainty stem from the cumulative impact scores, environmental component response to pressures, and literature-reported uncertainty on the CI model. The lowest rank is related to the EMODnet dataset for seabed habitats, human use data, land-based pollution and datasets for marine mammals, sea turtles and giant devil rays.

#### Global uncertainty analysis and sensitivity analysis (level 3)

**Fig 12:**
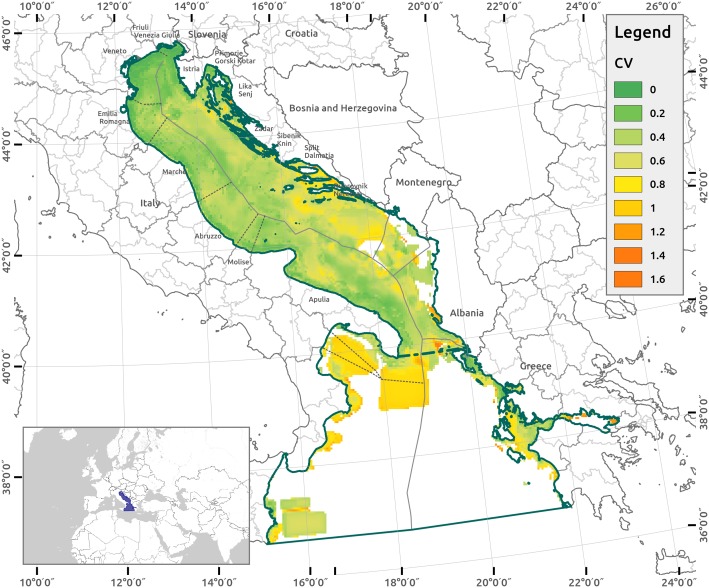
Uncertainty analysis of four input factors groups. The spatial distribution of the coefficient of variation expressed (CV)–resulting from the Monte Carlo simulation of the four input factors groups of i) sensitivity score errors, ii) pressure distance errors, iii) stressor combination factor and iv) response factor—is reported, from lower (dark green) to higher values (orange).

The uncertainty analysis shows the spatial variation of robustness in the CI assessment results. Fig 12 reports the spatial distribution of uncertainty for the four input factors considering the estimated coefficient of variation (CV) over each cell for the CI output. The CV values are generally lower in the Italian Adriatic and higher in the Ionian and Central East Adriatic (in front of Southern Croatia, Montenegro and in the Strait of Otranto between Italy and Albania).

**Fig 13:**
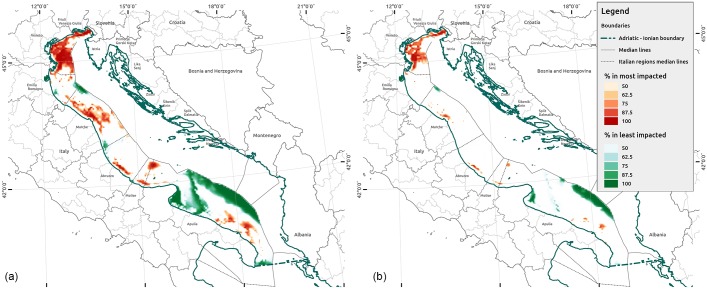
High- and low-impact areas according to the results of 50% to 100% of the Monte Carlo simulations in the Italian Adriatic area. The maps show the percentage of how often each grid cell was in the most and least impacted 25% (a) and 10% (b) of the Italian Adriatic region. The red gradient refers to most impacted, the green one to least impacted for the percentage (between 50% to 100%) of simulation runs.

Fig 13 compares high- and low-impact areas according to the results of the Monte Carlo simulations in the Italian Adriatic area, which is covered by complete and homogeneous input data according to the level 1 analysis. In these areas, the CI scores are stable around similar values (high or low), and the uncertainty CV is lower. The area that falls within the most impacted 25% of scores and in at least 50% of simulation runs covers 23% of the Italian Adriatic (approximately 14,800 km^2^) (S5 Fig). Thus, the 5% of the Italian Adriatic was ranked among of the most impacted 25% scores in at least 95% of the simulation runs. This percentage drops to 1.9% if we consider the most impacted 10% scores. The areas that fall in the least impacted 25% scores in at least 50% of the simulation runs cover 25% of the Italian Adriatic (approximately 16,100 km^2^) (S5 Fig). This percentage drops to 1.7% if we consider the least impacted 10% (S5 Fig).

**Fig 14:**
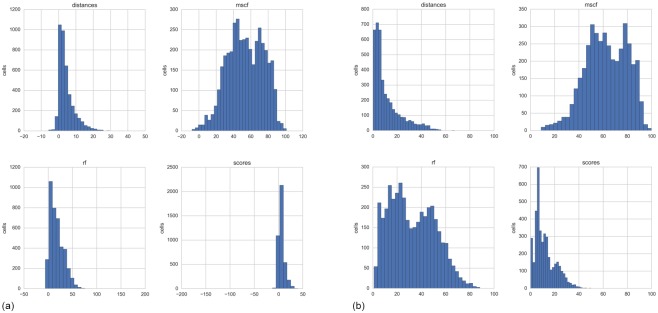
Distribution of first-order index (a) and total-effect index (b) for each grid cell.

With respect to the sensitivity analysis, the first-order and total-effect sensitivity measures for the four input factors are estimated for each grid cell. The results, reported in Table 4, show that (on average) the most important factor determining uncertainty is the multi-stressor combination factor (mscf), with a mean first-order sensitivity of 53.6%, followed by the nonlinear response factor (rf) of 17.5%. The sensitivity score errors and the pressure distance errors are the least problematic with a 5.9% and 4.4% individual (on average) contribution to the output uncertainty, respectively. As shown in Fig 14A, the first-order indices can highly vary across the cells of the analysis region, especially for the msf and rf input factors, where the respective histograms have high dispersion and variability.

**Table 4:** Mean values of the first order index (S1) and total order index (ST).

Factor groups	S1 mean	ST mean
**mscf**	53.6	62.3
**rf**	17.5	32.6
**scores**	5.9	12.1
**distances**	4.4	11.7

The values are expressed in percentage.

**Fig 15:**
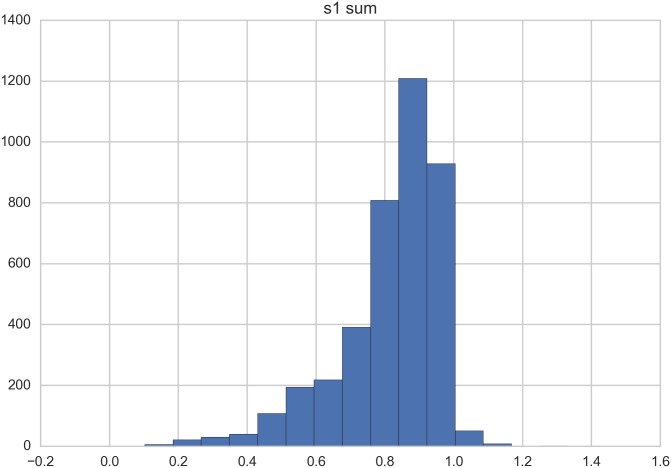
Distribution of sums of first order indexes.

The sum of the first-order indices (Table 4, S1 mean) is approximately 81.4%, indicating that, on average, there is some interaction (18.6%) between the inputs (the complete absence of interaction would produce a sum of 100%). The variability of the first-order’s sum across the grid cells is shown in Fig 15. With respect to the second-order sensitivity measure, in Table 5, the mean value for each pair combination of factors is reported. The rf factor shows a higher interactions with all of the other factors.

**Table 5:** Mean value of second-order interaction between pair of input factors.

**Factor groups**	**mscf**	**rf**	**distances**	**scores**
**mscf**	-	3.1	0	0.7
**rf**	3.1	-	3.8	1.0
**distances**	0	3.8	-	0
**scores**	0.7	1.0	0	-

The values are expressed in percentage.

## Discussion

### Method for general uncertainty analysis

The method for uncertainty analysis divided into three levels enables identification of the sources of uncertainties that might derive from the peculiarities of regional case studies. This uncertainty was a limitation mentioned in Stock and Micheli [8], who suggested performing an uncertainty analysis related to the specific case study areas to detect region-based sources of uncertainty.

The construction of the uncertainty matrix leads to the co-production of knowledge on the best available science and uncertainty on the case study area. This process is managed by the modelers with insights and revision from the community of experts and stakeholders who take part in the MSP process. This assures the positive inclusion of multiple sources of knowledge from diverse entities, which benefits the MSP process [60–63]. Inclusion of the qualitative and quantitative methods within the 3-level analysis allows for integrating expert elicitation and stakeholders’ observations. A limit of this method is that it is performed on a voluntary basis, especially with regard to the expert survey, and the perception of the experts and stakeholders is a real limiting factor in the solidity and statistical validity of the results [64,65]. Being aware of such limits, the uncertainty and sensitivity analysis include the score errors factor to test the robustness of the output based on the variation of scores. Moreover, we confronted the baseline run with the results of the uncertainty and sensitivity analysis to verify the relevance of the uncertainty derived from the expert survey and stakeholders’ inputs, namely, the sensitivity scores and the distance models.

The three-level method is structured to guide modelers in understanding and recording uncertainty in its multiple sources. Moreover, the method allows us to transparently identify the areas where global numerical uncertainty and sensitivity analysis can be applied without sacrificing inconsistencies or deformations of the results due to data gaps. For example, extending the analysis to the areas where we have no data regarding uses may underestimate the influence of scores and distances factors in the global uncertainty. The level 1 and 2 analysis covers the entire case study area (AIR), as well as the uncertainty (UA) in level 3, while the sensitivity analysis (SA) covers only the Italian Adriatic region because missing input data will influence the sensitivity ranking connected to the scores’ errors, as suggested by Stock and Micheli [8]. Integration of the results from the 3 levels of analysis can help identify uncertainty concerns for the entire case study area.

Considering the quantitative analysis of level 3, advantages of the Sobol (and extensions) method [43] include, along with other methods based on the decomposition of variance, ensuring that the entire model input space is explored and that the method is a model-free sensitivity measure, which is independent of assumptions about the model structure (e.g., when the model is non-linear and non-monotonic). Other methods can be of limited use, if not outright misleading, when the analysis assesses the relative importance of model inputs [48]. Additionally, this approach distinguishes between the first-order effects and higher-order effects that account for the interactions. Such information can be useful for model improvement, parameter estimation, or model simplification. Furthermore, as shown by Tang et al. [49], the method yields more robust sensitivity rankings than other measures such as analysis of variance or regional sensitivity analysis [50].

### General uncertainty analysis for the MSP

This study provides an operative tool to evaluate the uncertainty included in CI assessments in relation to the MSP general framework and process and applies it to a case study in the AIR. As MSP is meant to be an adaptive process based on the best available knowledge, the method depicts the most important sources of uncertainty on which to focus in subsequent planning cycles.

The method can be used to support decision makers in negotiating the CI risk acceptability with stakeholders and to identify thresholds of “acceptable uncertainty” when exploring uncertainty ranges of variability. Notably, this study provides a method to support and inform decision makers while verifying that the three following conditions to claim the precautionary principle are satisfied: i) the potentially adverse effects are identified, ii) the availability of scientific data is evaluated, and iii) the extent of scientific uncertainty is analyzed [4]. Along with the 3 conditions, we highlighted some caveats the decision-makers could face while setting the uncertainty analysis for MSP.

With respect to condition 1 listed above, we identified the areas where potentially adverse effects might occur with the baseline run of the CI assessment model and the 15,000 simulation runs (Figs 5 and 13) for impacts higher than 25% according to previous studies [8,52]. With respect to condition 2, we analyzed scientific data availability considering all of the uncertainty locations and sub-locations required by the CI model in the level 1 and 2 analysis. With respect to condition 3, we quantitatively and qualitatively analyzed the extent of scientific uncertainty in the case study area, considering the spatial variation on the grid cells.

The method depicts the range of uncertainty variability for the level 3 uncertainty analysis, where the CV varies from 0.0 to 1.6. However, the CI uncertainty threshold, which entails the related risk in producing impacts, is not pre-determined or identified by law. The level of risk acceptability connected with the level of CI uncertainty should be the result of “an eminently political decision” (COM (2000) 1 final, p. 15 [4]) considered by the authorities involved in the MSP process.

Moreover, with respect to the level of impacts, Halpern et al. [52] considers the greatest 25% of the impacts as the significant warning threshold. With the sensitivity analysis, we depicted the frequencies of each grid cell to show the impacts above the 25^th^ percentile with respect to the MC simulation runs (15,000) (S5 Fig). We found that 5% of the Italian Adriatic was ranked among of the most impacted 25% of the scores in at least 95% of the simulation runs. This implies that, even varying the model hypothesis, these areas will be highly impacted. Consequently, we can affirm with 95% confidence that the CI will be high. For these areas, the MSP should act to reduce and control the CI, without needing to claim the precautionary principle. Conversely, a significant number of cells show impacts greater than the 25^th^ percentile for a lower number of simulation runs (S5 Fig). For example, 40% of the Italian Adriatic is impacted over 25% in at least 15% of the CI simulation runs. This implies that mechanisms of multiple stressors need to be further investigated in these areas, where uncertainty is very significant and CI scores vary significantly under different hypotheses. Moreover, in all these other cases, the (political) definition of the acceptable risk will identify the frequency threshold related to the potential CI probability. According to this threshold, areas where it would be necessary to claim the precautionary principle can be identified. In Fig 15, we assumed a threshold of 50% of the simulation runs, considering a probability of 0.5 of the greatest CI event (CI>25^th^ percentile); but a more conservative approach could have considered probabilities of 30% or 25%. The definition of thresholds should follow the general decision of acceptable risk as a prominent activity of the MSP process. This methodology allows one to verify and test the extent of the areas under different risk thresholds hypothesized along with the decision-making process.

In order to fully define the scientific and data uncertainty, the method allows identification of all of the different sources of uncertainties, not only the ones that can be quantified statistically (with consolidated techniques as an uncertainty and sensitivity analysis [8,48,50], which are basically related to the model algorithm and its capacity to represent the phenomenon of concern). In the level 1 analysis, the method classifies the sources of uncertainty and relates them to the assumptions and constraints directly derived from the MSP framework for which the CI assessment is built. For example, an important source of uncertainty resides in the sub-location of the spatial context of the analysis that directly influences all of the other sources, especially for the input uncertainties related to the input data gaps and knowledge gaps. In our study, the geographical scope of the analysis is policy-driven and not model-driven, meaning that the geographical scope was established by the MSP process under the EUSAIR, including areas with significant input data gaps or environmental components with very limited scientific knowledge (as with the deep sea). This is in contrast to the work of Korpinen et al. [16], who decided to limit the case study area when running the CI model to avoid inconsistencies and data gaps.

During the real MSP process, the geographical scope is defined by the MSP mandate and is usually derived from the domain of responsibility of the institutions involved in the plan. This study demonstrates that during the real decision-making processes, modelers are confronted and constrained to set their model according to the planning domain, which very rarely corresponds to the optimal domain where to run models (i.e., with complete and harmonized input data, and with solid knowledge on environmental dynamics). While strategies to cover these gaps include the use of proxies [8] or surrogates [66,67], performing the general uncertainty analysis to clearly communicate the limitations of the CI assessment results is important for decision makers and planners, as well as the sources of those limitations. Our results show a variety of sources of uncertainty that are related to the MSP framework, and they are mentioned in the uncertainty matrix (level 1 of the uncertainty analysis) and play an important role in the uncertainty ranking per sub-location (level 2 of the uncertainty analysis). In fact, “the geographical domain of CI analysis” is the most significant in terms of scenario uncertainty—the variability is not statistically determined but is inherent to the MSP process and the way the countries will implement their marine spatial plans (for example, the geographical scope and cross-border sources of impacts).

We propose a Data Availability Index (DAI) to support the spatial characterization of input uncertainty (level 1 uncertainty analysis) as it detects the data gaps occurring due to the specific geographical scope of analysis. The DAI is a geospatial screening tool used by modelers to identify the geographic locations for the preliminary sophisticated uncertainty technique deployment, performed in level 3. Similarly, the local sensitivity confidence index (LSCI) performs a geospatial analysis on the sensitivity confidence scores at an early stage of the modelling process that can be used for effective communication of the results for decision-makers and planners involved in the MSP process. The LSCI spatial explicit information can be used to look for the best available knowledge necessary to clarify the potential sources of uncertainties, and in any case, to fill the knowledge gaps occurring along the planning process. Therefore, the DAI and LSCI are rapid indicators calculated with a simple, analytic design that is already included in the CI model. The level 2 analysis is meant to synthetically communicate the importance (i.e., weight) of the various sources of uncertainties to the planners and decision makers to take due actions with the stakeholders or experts involved in the MSP process to cover those limitations and gaps where possible.

To explore the scientific uncertainty, the analysis prioritizes identifying sources of uncertainty that mostly influenced the CI assessment in the AIR. Level 2 denotes the sub-location of sensitivities (as a combination of the uncertainty levels of statistical nature, the scenario, the recognized ignorance, the variability and the epistemic nature). The level 3 details and quantifies factors related to the sensitivity model uncertainty, which is the most influential of all the uncertainty locations. Moreover, the integration of level 2 and 3 allows one to characterize the aspects of the sensitivities that are the most urgent. From level 1 and 2, depicting the environmental components (and where they are) in the AIR that are less known by experts is possible, which is mostly the sensitivities of seabirds, deep sea habitats and some seabed habitats (A5.39, A5.46, A5.36). Moreover, in level 1, these knowledge gaps are spatially located (the LSCI red areas in Fig 9). As determined by the level 3 uncertainty analysis, the robustness in the CI scores varies significantly between the grid cells, just as the robustness also emerges from the LSCI variability. The analysis demonstrates the local variability of uncertainty, which is depicted as a fine scale.

As determined by the level 3 sensitivity analysis performed for the Italian Adriatic, the multi-stressor combination factor (mscf) and the nonlinear response factor (rf) are the dominant factors in determining the CI scores’ uncertainty compared to the sensitivity scores and the distance models. Moreover, the factors’ great influence is largely distributed across the cells (Fig 14), meaning that the uncertainty is a significant concern in the CI model in large areas of the Italian Adriatic. To gain better knowledge on the mscf and rf mechanisms, improving scientific research on the response of the ecosystems to anthropogenic pressures and the related sensitivity mechanisms is necessary. The research priority should focus on the sensitivities with a high contribution to the CI scores and the sensitivities with a high uncertainty level (low confidence from science). Presently, most of the contributing sensitivities emerging from the baseline run (reported in S4 Fig) are related to trawling and maritime transport with respect to the nursery areas, seabed habitat A5.39 and seabirds. Moreover, the specific sensitivity analysis could be performed to rank the importance of the sensitivities within the factors used in the level 3 analysis; however; this was not performed in our method due to computational limitations between the number of factors (4) and the number of MC simulation runs (15,000). Basic research in marine ecology is moving towards a better understanding of the cumulative effects of multiple pressures, regime shifts and resilience in marine ecosystems [56–58]. The uncertainty deriving from the application of cumulative, dominant or multiplicative models in the CI assessment, at stake with the mscf factor, is discussed in the literature [7,8], but the uncertainty is inherent to the present state of the art, as demonstrated in the case study.

## Conclusions

This study proposed a framework to incorporate the uncertainty of different sources while setting and implementing a CI assessment model for MSP, integrating expert judgement and elicitation to arrive at a complete (qualitative and quantitative) description of uncertainty. The three-level method can be implemented for other decision-making processes where a spatial description of uncertainty is required to elaborate informed spatial decisions related to human uses and potential stressors on the environment, as decision-making should consider the limitations related to the uncertainty aspects instead of hiding them [13, 68].

This paper addresses a major challenge in CI assessment, which is related to understanding relevant insights derived from the CI assessment considering all of the potential types of limitations and gaps emerging from uncertainty analysis. The proposed methodology contributes to understanding the response of environmental components on the combination of multiple threats of human activities, combining the geospatial distribution and intensity of the impacts to the geospatial distribution of uncertainty.

While this study demonstrates the necessity to strengthen the dialogue between science and policy to update knowledge to fill the gaps in policy needs, the ultimate decision on applying the precautionary principle—included in the definition of the acceptable risk—is predominantly a political decision. The general uncertainty analysis proposed in this study explores the extent of spatial variation of the acceptable risk of the potential CI, which might be defined through the thresholds and limits as emerged from the MSP process.

## Supporting information

S1 Fig Behaviours of sensitivity score errors (SC) and pressure distance errors (D) factors curves. For factors sensitivity score errors (SC) and pressure distance errors (D), we adopted the beta-distribution (B(s, c)) assuming the modal values from expert judgment on sensitivities (s), and the variance from the confidence (c).
(DOCX)

S2 Fig Data coverage of environmental components (E) and human uses (U). Data coverage is mapped for a. environmental components (E) and b. human uses (U) for the AIR, from which the Data Availability Index (DAI) is calculated. Dark blue indicates that all dataset are available, while light blue to red means that not all datasets are available.
(DOCX)

S3 Fig Classes of local sensitivity confidence index (LSCI) in the AIR. Values of LSCI are grouped from 0.8–1.0, which represent the maximum confidence expressed by expert judgement and 0.2–0.4, which represents the minimum confidence.
(DOCX)

S4 Fig Ranking sensitivity scores with respect to their contribution and weight for the AIR in the CI model output. The 60 sensitivity scores which contribute the most in the AIR are ranked according to the their total score; the confidence related to the EUP associated score expressed by the experts is reported as well.
(DOCX)

S5 Fig Percentage of the Italian Adriatic region area for the least (a) and most (b) impacted area over the number of Monte Carlo simulations. The x-axis represents how often each cell was in the least or most impacted area (% of simulations).
(DOCX)

S1 Table List of pressures according to Marine Strategy Framework Directive (MSFD, 2008/56/EC), annex III, tab. 2 (DOCX)

S2 Table List of human uses and related datasets for the CI calculation. The list of human uses considered for the CI is correlated with the data owners and data providers acronyms.
(DOCX)

S3 Table List of environmental components and related datasets for the CI calculation. The list of environmental components considered for the CI is correlated with the data owners and data providers acronyms.
(DOCX)

S4 Table Criteria evaluated by experts to express the EUP sensitivities. Criteria used within the expert survey to calculate the sensitivity score of environmental components to pressures deriving from maritime uses, adapted from Andersen et al. (2013).
(DOCX)

S5 Table Locations and sub-locations of uncertainty identified for the CI assessment model. Locations are identified according to Walker et al. 2003. Sub-locations are identified by the modellers within the level 1 of the general uncertainty analysis.
(DOCX)

S6 Table Cumulative impacts uncertainty matrix for the AIR. The subjects who have declared the different sub-locations, levels and nature of uncertainty, are indicated as M = modelers (authors of the paper), SE = stakeholders and experts (through interviews, workshops and survey), L = literature.
(DOCX)

S7 Table Data availability per human uses U for different geographical areas. (DOCX)

S1 File Gazetteer of spatial coverage of input dataset. A vocabulary of geographical areas was composed to characterize the geographical scope of each dataset. It consists of 22 terms, which can be modified and new terms can be added. Each term was associated to a specific geometry and related boundary, which was input in the model to calculate the data availability index (DAI).
(DOCX)
