# Supplementary material for: Addressing uncertainty in modelling cumulative impacts within maritime spatial planning in the Adriatic and Ionian region
Source: PLoS One. 2017 Jul 10;12(7):e0180501. doi: 10.1371/journal.pone.0180501 (PMC5503246; doi:10.1371/journal.pone.0180501)
Supplement: S1 Fig — For factors sensitivity score errors (SC) and pressure distance errors (D), we adopted the beta-distribution (B(s, c)) assuming the modal values from expert judgment on sensitivities (s), and the variance from the confidence (c). (DOCX) [file pone.0180501.s001.docx]

**S1 Fig. Behaviours of sensitivity score errors (SC) and pressure distance errors (D) factors curves.** For factors sensitivity score errors (SC) and pressure distance errors (D), we adopted the beta-distribution (B(s, c)) assuming the modal values from expert judgment on sensitivities (s), and the variance from the confidence (c).

| **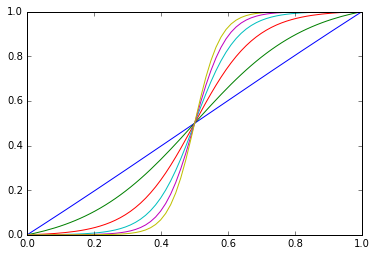** (s) curve |
| --- |
| **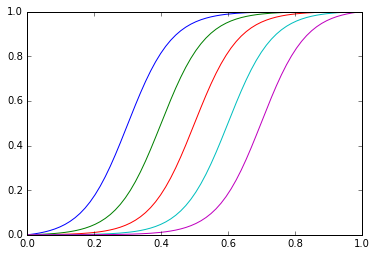** (c) curve |
