# Supplementary material for: Addressing uncertainty in modelling cumulative impacts within maritime spatial planning in the Adriatic and Ionian region
Source: PLoS One. 2017 Jul 10;12(7):e0180501. doi: 10.1371/journal.pone.0180501 (PMC5503246; doi:10.1371/journal.pone.0180501)
Supplement: S2 Fig — Data coverage is mapped for a. environmental components (E) and b. human uses (U) for the AIR, from which the Data Availability Index (DAI) is calculated. Dark blue indicates that all dataset are available, while light blue to red means that not all datasets are available. (DOCX) [file pone.0180501.s002.docx]

**S2 Fig. Data coverage of environmental components (E) and human uses (U).** Data coverage is mapped for a. environmental components (E) and b. human uses (U) for the AIR, from which the Data Availability Index (DAI) is calculated. Dark blue indicates that all dataset are available, while light blue to red means that not all datasets are available.

| 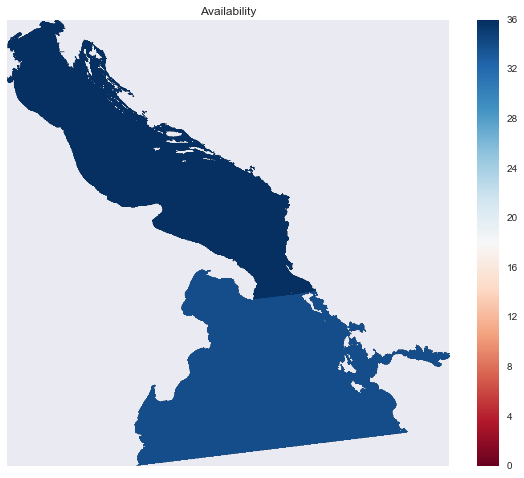  a. | 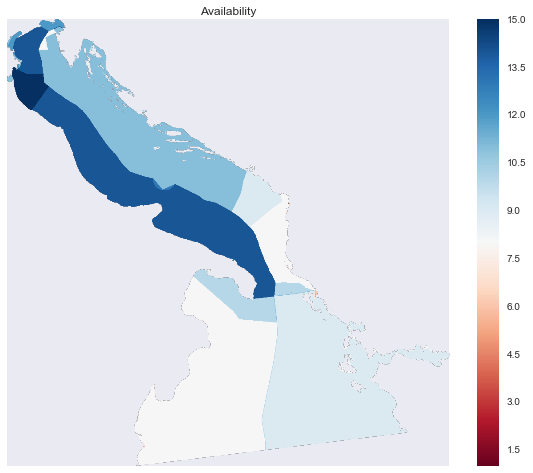  b. |
| --- | --- |
