# Supplementary material for: Addressing uncertainty in modelling cumulative impacts within maritime spatial planning in the Adriatic and Ionian region
Source: PLoS One. 2017 Jul 10;12(7):e0180501. doi: 10.1371/journal.pone.0180501 (PMC5503246; doi:10.1371/journal.pone.0180501)
Supplement: S3 Fig — Values of LSCI are grouped from 0.8–1.0, which represent the maximum confidence expressed by expert judgement and 0.2–0.4, which represents the minimum confidence. (DOCX) [file pone.0180501.s003.docx]

**S3 Fig. Classes of local sensitivity confidence index (LSCI) in the AIR.** Values of LSCI are grouped from 0.8-1.0, which represent the maximum confidence expressed by expert judgement and 0.2-0.4, which represents the minimum confidence.

| 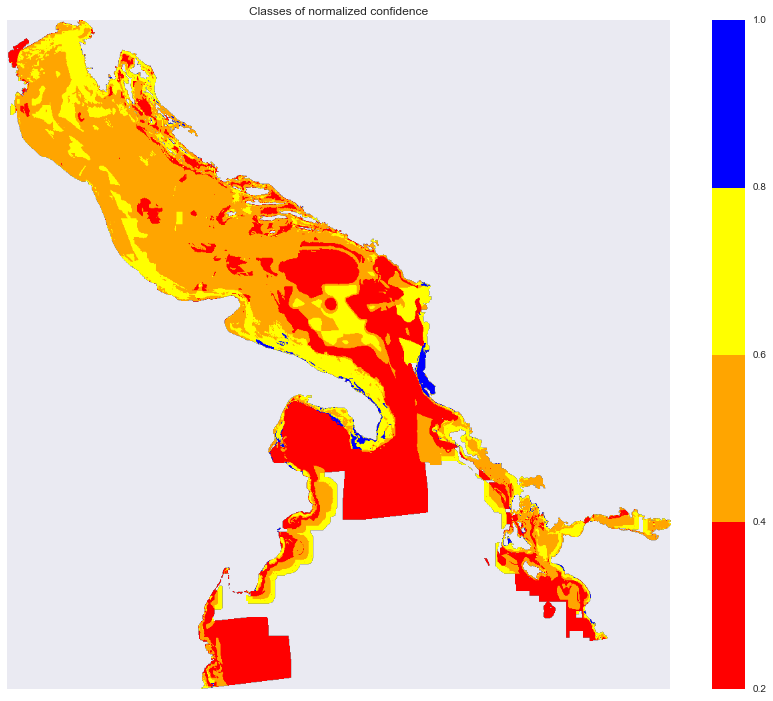 |
| --- |
