# Supplementary material for: Addressing uncertainty in modelling cumulative impacts within maritime spatial planning in the Adriatic and Ionian region
Source: PLoS One. 2017 Jul 10;12(7):e0180501. doi: 10.1371/journal.pone.0180501 (PMC5503246; doi:10.1371/journal.pone.0180501)
Supplement: S4 Fig — The 60 sensitivity scores which contribute the most in the AIR are ranked according to the their total score; the confidence related to the EUP associated score expressed by the experts is reported as well. (DOCX) [file pone.0180501.s004.docx]

**S4 Fig. Ranking sensitivity scores with respect to their contribution and weight for the AIR in the CI model output.** The 60 sensitivity scores which contribute the most in the AIR are ranked according to the their total score; the confidence related to the EUP associated score expressed by the experts is reported as well.

| **Rank** | **Human use (U)** | **Env. component (E)** | **Pressure (P)** | **score** | **confidence** |
| --- | --- | --- | --- | --- | --- |
| **1** | Trawling | NH - Nursery habitats | Marine litter | 650.145858586 | 0.600000 |
| **2** | Trawling | NH - Nursery habitats | Selective extraction of species, including inc... | 565.007710438 | 0.666667 |
| **3** | Trawling | A5.39 - Mediterranean biocenosis of coastal te... | Smothering | 435.68025576 | 0.600000 |
| **4** | Trawling | A5.39 - Mediterranean biocenosis of coastal te... | Changes in siltation | 393.007416607 | 0.600000 |
| **5** | Trawling | SB - Seabirds | Generic pressure 1 | 332.828862915 | 0.200000 |
| **6** | Trawling | SB - Seabirds | Generic pressure 2 | 332.828862915 | 0.200000 |
| **7** | Trawling | A5.36 - Circalittoral fine mud | Changes in siltation | 317.888425855 | 0.600000 |
| **8** | Trawling | A5.46 - Mediterranean biocenosis of coastal de... | Selective extraction of species, including inc... | 315.201470027 | 0.600000 |
| **9** | Trawling | A5.46 - Mediterranean biocenosis of coastal de... | Abrasion (surface, light, heavy) | 315.201470027 | 0.400000 |
| **10** | Maritime Transport | A5.46 - Mediterranean biocenosis of coastal de... | Marine litter | 311.920088953 | 0.400000 |
| **11** | Maritime Transport | TU - Turtles | Marine litter | 306.623459652 | 0.800000 |
| **12** | Maritime Transport | TU - Turtles | Underwater noise | 257.563706107 | 0.800000 |
| **13** | Military Areas | BDS - Bathypelagic component of deep sea areas | Generic pressure 1 | 236.416232872 | 0.200000 |
| **14** | Military Areas | BDS - Bathypelagic component of deep sea areas | Generic pressure 2 | 236.416232872 | 0.200000 |
| **15** | Trawling | A5.35 - Circalittoral sandy mud | Smothering | 231.636010621 | 0.400000 |
| **16** | Trawling | A5.35 - Circalittoral sandy mud | Abrasion (surface, light, heavy) | 231.636010621 | 0.400000 |
| **17** | Maritime Transport | MM - Marine mammals | Underwater noise | 224.51296925 | 0.666667 |
| **18** | Trawling | A4.26 - Mediterranean coralligenous communities | Changes in siltation | 212.58 | 1.000000 |
| **19** | Small scale fishery | A4.26 - Mediterranean coralligenous communities | Abrasion (surface, light, heavy) | 196.1325 | 0.900000 |
| **20** | Maritime Transport | A4.26 - Mediterranean coralligenous communities | Introduction of non-indigenous species and tra... | 194.17001625 | 1.000000 |
| **21** | Maritime Transport | A4.26 - Mediterranean coralligenous communities | Introduction of non-synthetic substances and c... | 194.17001625 | 1.000000 |
| **22** | Trawling | A4.26 - Mediterranean coralligenous communities | Abrasion (surface, light, heavy) | 192.503 | 0.840000 |
| **23** | Maritime Transport | BDS - Bathypelagic component of deep sea areas | Generic pressure 2 | 162.744440552 | 0.200000 |
| **24** | Maritime Transport | BDS - Bathypelagic component of deep sea areas | Generic pressure 1 | 162.744440552 | 0.200000 |
| **25** | Trawling | A5.36 - Circalittoral fine mud | Abrasion (surface, light, heavy) | 161.880743213 | 0.600000 |
| **26** | Small scale fishery | A4.26 - Mediterranean coralligenous communities | Selective extraction of species, including inc... | 152.963072846 | 0.640000 |
| **27** | Maritime Transport | MM - Marine mammals | Marine litter | 148.069533649 | 0.800000 |
| **28** | Maritime Transport | SB - Seabirds | Generic pressure 2 | 147.956695194 | 0.200000 |
| **29** | Maritime Transport | SB - Seabirds | Generic pressure 1 | 147.956695194 | 0.200000 |
| **30** | Small scale fishery | A5.23 - Infralittoral fine sands | Marine litter | 134.681733896 | 0.400000 |
| **31** | Coastal and Maritime Tourism | A4.26 - Mediterranean coralligenous communities | Abrasion (surface, light, heavy) | 130.209707066 | 0.900000 |
| **32** | Oil & Gas research | BDS - Bathypelagic component of deep sea areas | Generic pressure 2 | 119.207576923 | 0.200000 |
| **33** | Oil & Gas research | BDS - Bathypelagic component of deep sea areas | Generic pressure 1 | 119.207576923 | 0.200000 |
| **34** | Maritime Transport | A5.46 - Mediterranean biocenosis of coastal de... | Underwater noise | 118.142392855 | 0.400000 |
| **35** | Small scale fishery | SB - Seabirds | Generic pressure 1 | 116.984131848 | 0.200000 |
| **36** | Small scale fishery | SB - Seabirds | Generic pressure 2 | 116.984131848 | 0.200000 |
| **37** | Trawling | TU - Turtles | Selective extraction of species, including inc... | 116.456232226 | 0.800000 |
| **38** | Maritime Transport | A6.511 - Facies of sandy muds with Thenea muri... | Generic pressure 1 | 116.04384775 | 0.200000 |
| **39** | Maritime Transport | A6.511 - Facies of sandy muds with Thenea muri... | Generic pressure 2 | 116.04384775 | 0.200000 |
| **40** | Oil & Gas research | A6.51 - Meditteranean communities of bathyal muds | Generic pressure 2 | 106.418209164 | 0.200000 |
| **41** | Oil & Gas research | A6.51 - Meditteranean communities of bathyal muds | Generic pressure 1 | 106.418209164 | 0.200000 |
| **42** | Coastal and Maritime Tourism | A4.26 - Mediterranean coralligenous communities | Selective extraction of species, including inc... | 106.09679835 | 1.000000 |
| **43** | Trawling | A6.51 - Meditteranean communities of bathyal muds | Smothering | 101.015596757 | 0.600000 |
| **44** | Trawling | A5.26 - Circalittoral muddy sand | Smothering | 99.6260465272 | 0.600000 |
| **45** | Maritime Transport | A5.47 - Mediterranean biocenosis of shelf-edge... | Marine litter | 98.3309893063 | 0.400000 |
| **46** | Trawling | BDS - Bathypelagic component of deep sea areas | Generic pressure 2 | 96.1471076581 | 0.200000 |
| **47** | Trawling | BDS - Bathypelagic component of deep sea areas | Generic pressure 1 | 96.1471076581 | 0.200000 |
| **48** | Trawling | A5.38 - Mediterranean biocenosis of muddy detr... | Changes in siltation | 90.6943938036 | 0.800000 |
| **49** | Trawling | A5.47 - Mediterranean biocenosis of shelf-edge... | Abrasion (surface, light, heavy) | 90.5938854965 | 0.400000 |
| **50** | Trawling | A5.47 - Mediterranean biocenosis of shelf-edge... | Selective extraction of species, including inc... | 90.5938854965 | 0.600000 |
| **51** | Small scale fishery | A5.46 - Mediterranean biocenosis of coastal de... | Generic pressure 1 | 81.5727098766 | 0.200000 |
| **52** | Small scale fishery | A5.46 - Mediterranean biocenosis of coastal de... | Generic pressure 2 | 81.5727098766 | 0.200000 |
| **53** | Coastal and Maritime Tourism | A5.23 - Infralittoral fine sands | Changes in siltation | 74.4769416706 | 0.600000 |
| **54** | Trawling | GDR - Giant devil ray | Generic pressure 2 | 74.0653078033 | 0.200000 |
| **55** | Trawling | GDR - Giant devil ray | Generic pressure 1 | 74.0653078033 | 0.200000 |
| **56** | Aquaculture | A5.23 - Infralittoral fine sands | Introduction of non-indigenous species and tra... | 71.1536350844 | 0.400000 |
| **57** | Trawling | A5.26 - Circalittoral muddy sand | Abrasion (surface, light, heavy) | 69.738232569 | 0.600000 |
| **58** | Trawling | MM - Marine mammals | Selective extraction of species, including inc... | 68.3555257755 | 0.800000 |
| **59** | Trawling | A6.51 - Meditteranean communities of bathyal muds | Selective extraction of species, including inc... | 68.0297503905 | 0.800000 |
| **60** | Small scale fishery | A5.535 - Posidonia beds | Introduction of non-indigenous species and tra... | 67.9474525448 | 0.400000 |
