# Supplementary material for: Addressing uncertainty in modelling cumulative impacts within maritime spatial planning in the Adriatic and Ionian region
Source: PLoS One. 2017 Jul 10;12(7):e0180501. doi: 10.1371/journal.pone.0180501 (PMC5503246; doi:10.1371/journal.pone.0180501)
Supplement: S5 Fig — The x-axis represents how often each cell was in the least or most impacted area (% of simulations). (DOCX) [file pone.0180501.s005.docx]

**S5 Fig. Percentage of the Italian Adriatic region area for the least (a) and most (b) impacted area over the number of Monte Carlo simulations.** The x-axis represents how often each cell was in the least or most impacted area (% of simulations).

| **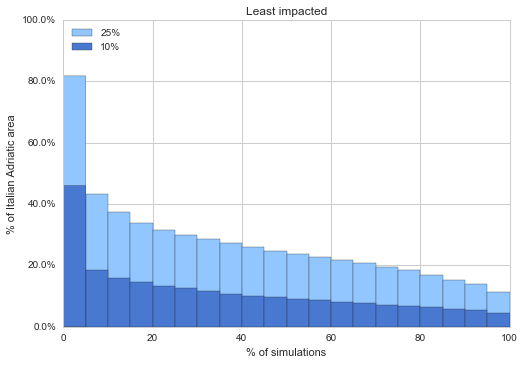**  a. | **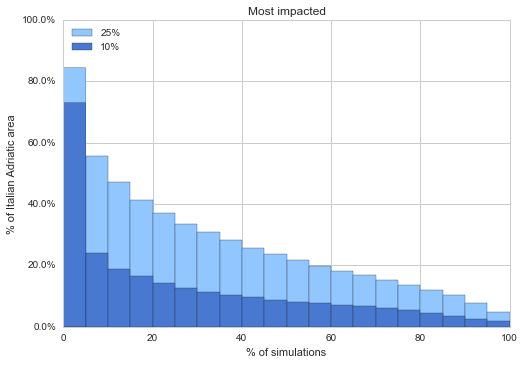**  b. |
| --- | --- |
