# Supplementary material for: Addressing uncertainty in modelling cumulative impacts within maritime spatial planning in the Adriatic and Ionian region
Source: PLoS One. 2017 Jul 10;12(7):e0180501. doi: 10.1371/journal.pone.0180501 (PMC5503246; doi:10.1371/journal.pone.0180501)
Supplement: S1 Table — (DOCX) [file pone.0180501.s006.docx]

**S1 Table. List of pressures according to Marine Strategy Framework Directive (MSFD, 2008/56/EC), annex III, tab. 2.**

| **Pressure** | **Description** |
| --- | --- |
| Physical loss | Smothering (e.g. by man-made structures, disposal of dredge spoil), |
|  | Sealing (e.g. by permanent constructions) |
| Physical damage | Changes in siltation (e.g. by outfalls, increased run-off, dredging/disposal of dredge spoil) |
|  | Abrasion (e.g. impact on the seabed of commercial fishing, boating, anchoring) |
|  | Selective extraction (e.g. exploration and exploitation of living and non-living resources on seabed and subsoil) |
| Other physical disturbance | Underwater noise (e.g. from shipping, underwater acoustic equipment) |
|  | Marine litter |
| Interference with hydrological processes | Significant changes in thermal regime (e.g. by outfalls from power stations) |
|  | Significant changes in salinity regime (e.g. by constructions impeding water movements, water abstraction) |
| Contamination by hazardous substances | Introduction of synthetic compounds (e.g. priority substances under Directive 2000/60/EC which are relevant for the marine environment such as pesticides, antifoulants, pharmaceuticals, resulting, for example, from losses from diffuse sources, pollution by ships, atmospheric deposition and biologically active substances) |
|  | Introduction of non-synthetic substances and compounds (e.g. heavy metals, hydrocarbons, resulting, for example, from pollution by ships and oil, gas and mineral exploration and exploitation, atmospheric deposition, riverine inputs) |
|  | Introduction of radio-nuclides |
| Systematic and/or intentional release of substances | Introduction of other substances, whether solid, liquid or gas, in marine waters, resulting from their systematic and/or intentional release into the marine environment, as permitted in accordance with other Community legislation and/or international conventions. |
| Nutrient and organic matter enrichment | Inputs of fertilisers and other nitrogen and phosphorus-rich substances (e.g. From point and diffuse sources, including agriculture, aquaculture, atmospheric deposition) |
|  | Inputs of organic matter (e.g. sewers, mariculture, riverine inputs) |
| Biological disturbance | Introduction of microbial pathogens |
|  | Introduction of non-indigenous species and translocations |
|  | Selective extraction of species, including incidental non-target catches (e.g. by commercial and recreational fishing) |

**References**

European Commission (2008), Framework Directive 2008/56/EC (MSFD) on the Strategy for the Marine Environment.
