# Supplementary material for: Addressing uncertainty in modelling cumulative impacts within maritime spatial planning in the Adriatic and Ionian region
Source: PLoS One. 2017 Jul 10;12(7):e0180501. doi: 10.1371/journal.pone.0180501 (PMC5503246; doi:10.1371/journal.pone.0180501)
Supplement: S2 Table — The list of human uses considered for the CI is correlated with the data owners and data providers acronyms. (DOCX) [file pone.0180501.s007.docx]

**S2 Table. List of human uses and related datasets for the CI calculation.** The list of human uses considered for the CI is correlated with the data owners and data providers acronyms.

| **Human uses** | **Description** | **Type of data^+^** | **Data format** | **Spatial coverage of the dataset*** | **Sources**** | **Data**  **Repository code°** |
| --- | --- | --- | --- | --- | --- | --- |
| Aquaculture | (e.g. marine farms and shellfish farms) | PA | V | Abruzzo, Adriatic Apulia, Croatia, Emilia Romagna, Greece, Marche, Molise, Montenegro, Slovenia, Veneto | Veneto Region, SHAPE, HCMR | u84 |
| Cables and Pipelines | (e.g. telecommunication cables, TAP) | PA | V | AIR | SHAPE, HCMR, OTE S.A. | u83 |
| Coastal and Maritime Tourism | (e.g. marinas and recreational boating) | I/PR | R (1 km) | Abruzzo, Apulia, Emilia Romagna, Greece, Marche, Molise, Slovenia, Veneto. | (modeled) | u76 |
| Coastal Defence Work | Marine infrastructures of coastal defense | PA | V | Abruzzo, Apulia, Emilia Romagna, Marche, Molise, Veneto | SHAPE, SIT Puglia Region | u78 |
| Dumping area for dredging | (e.g. dumping areas) | PA | V | Emilia Romagna | SHAPE | u90 |
| LNGs | Liquefied Natural Gas platforms | PA | V | AIR | OGS | u81 |
| Maritime Transport | (e.g. shipping, commercial traffic and cruise traffic) | I | R (250m) | Adriatic | CNR ISMAR | u75 |
| Military areas | Military infrastructures and training areas (Lawrence et al. 2015) | PA | V | Adriatic, Italian Ionian. | SHAPE, MIPAAF | u88 |
| Naval Based Activities | (e.g. cargo ports and maritime freight) | I/PR | R (1 km) | AIR | (modeled) | u77 |
| Off-shore sand deposit | (e.g. active sites of sand extraction) | PA | V | Italian Adriatic | CNR ISMAR, Regione Emilia Romagna, Regione Veneto, Arenaria S.r.l. | u89 |
| Oil and Gas Extraction | Concessions and platforms for exploitation of hydrocarbons and natural gas | PA | V | Italian Adriatic, Croatia | CHA, MEDTRENDS, MESMGR, MISE, SHAPE | u79 |
| Oil and Gas Research | Concessions for research and exploration on hydrocarbons and natural gas | PA | V | AIR | CHA, MEDTRENDS, MESMGR, MISE, SHAPE | u80 |
| Renewable Energy facilities | (e.g. off-shore wind farm) | PA | V | AIR | HCMR, OGS and RAE | u82 |
| Small scale Fishery | (e.g. it includes all fishing systems with gel netting and seine nets) | I | V | Italian Adriatic, Croatia, Ionian, Slovenia | CNR ISMAR | u87 |
| Trawling | Bottom and pelagic trawling (fishery) | I | R (1 km) | AIR | Blue Hub | u85 |

+ PA=presence/absence, I= intensity, where PR=proxy.

* Spatial coverage of datasets is described in supplementary materials S1 Text.

** Acronyms of data owners or data providers as Institutions or Projects are reported in a section here below titles “Data owners and data providers acronyms”

° Datasets are archived in the data repository at doi: 10.5281/zenodo.58222

**Data owners and data providers acronyms**

Arenaria S.r.l. www.arenariasabbie.com/

Blue Hub, JRC in-house platform to exploit big data in the maritime domain, https://bluehub.jrc.ec.europa.eu/

CHA, Croatian Hydrocarbons Agency, http://www.azu.hr/

CNR ISMAR, Consiglio Nazionale delle Ricerche, Istituto di Scienze Marine, www.ismar.cnr.it

HCMR, Hellenic Centre for Marine Research, www.hcmr.gr

MEDTRENDS, The Mediterranean Sea, Trends, Threats and Recommendations, http://www.medtrends.org/

MESMGR, Ministry of Economy, Sector for Mining and Geological Research, www.petroleum.me

MIPAAF, Italian Ministry of Agriculture, Food and Forests (Ministero delle Politiche Agricole, Alimentari e Forestali), https://www.politicheagricole.it/

MISE, Italian Ministry for Economic Development (Ministero per lo Sviluppo Economico), http://www.sviluppoeconomico.gov.it

OGS, Istituto Nazionale di Oceanografia e di Geofisica Sperimentale, http://www.ogs.trieste.it/it

OTE S.A. Hellenic Telecommunications Organisation, www.ripe.net

RAE, Regulatory Authority for Energy, www.rae.gr

Regione del Veneto, https://www.regione.veneto.it/

Regione Emilia Romagna, www.regione.emilia-romagna.it/

SHAPE, Shaping an Holistic Approach to Protect the Adriatic Environment between coast and sea, http://www.shape-ipaproject.eu/

SIT Apulia Region, www.sit.puglia.it/

**References**

Lawrence, M. J., Stemberger, H. L., Zolderdo, A. J., Struthers, D. P., & Cooke, S. J. (2015). The effects of modern war and military activities on biodiversity and the environment. Environmental Reviews, 23(4), 443-460.
