# Supplementary material for: Addressing uncertainty in modelling cumulative impacts within maritime spatial planning in the Adriatic and Ionian region
Source: PLoS One. 2017 Jul 10;12(7):e0180501. doi: 10.1371/journal.pone.0180501 (PMC5503246; doi:10.1371/journal.pone.0180501)
Supplement: S3 Table — The list of environmental components considered for the CI is correlated with the data owners and data providers acronyms. (DOCX) [file pone.0180501.s008.docx]

**S3 Table. List of environmental components and related datasets for the CI calculation.** The list of environmental components considered for the CI is correlated with the data owners and data providers acronyms.

| **Environmental component** | **Description** | **Type of data^+^** | **Data format** | **Spatial coverage of the dataset** | **Source°** | **Data**  **Repository code**^§^ |
| --- | --- | --- | --- | --- | --- | --- |
| **Seabed habitats** |  |  |  |  |  |  |
| A3- Infralittoral rock and other hard substrata | From EUNIS Marine Habitat classification (2007) | PA | V | AIR | EUNIS Marine Habitat classification 2007 (revised description 2012)** | e2 |
| A4.7 - Circalittoral rock and other hard substrata |  | PA | V | AIR |  | e3 |
| A4.26 - Mediterranean coralligenous communities |  | PR | V | AIR | MEDISEH-MAREA | e4 |
| A4.27 - Fauna communities on deep moderate energy |  | PA | V | AIR | EUNIS Marine Habitat classification 2007 (revised description 2012)** | e5 |
| A5.13 - Infralittoral coarse sediment |  | PA | V | AIR |  | e6 |
| A5.14 - Circalittoral coarse sediment |  | PA | V | AIR |  | e7 |
| A5.23 - Infralittoral fine sands |  | PA | V | AIR |  | e8 |
| A5.25 - Circalittoral fine sands |  | PA | V | AIR |  | e9 |
| A5.26- Circalittoral muddy sand |  | PA | V | AIR |  | e10 |
| A5.35 - Circalittoral sandy mud |  | PA | V | AIR |  | e11 |
| A5.36- Circalittoral fine mud |  | PA | V | AIR |  | e12 |
| A5.38- Mediterranean biocenosis of muddy detritic bottoms |  | PA | V | AIR |  | e13 |
| A5.39 - Mediterranean biocenosis of coastal terrigenous muds |  | PA | V | AIR |  | e14 |
| A5.46 - Mediterranean biocenosis of coastal detritic bottoms |  | PA | V | AIR |  | e15 |
| A5.47 - Mediterranean biocenosis of shelf-edge detritic bottoms |  | PA | V | AIR |  | e16 |
| A5.535- Posidonia beds |  | PR | V | AIR | MEDISEH-MAREA | e18 |
| A5.531 - Cymodocea beds |  | PA | V | AIR | EUNIS Marine Habitat classification 2007 (revised description 2012)** | e19 |
| A6.1 - Deep-sea rock and artificial hard substrata |  |  |  |  |  | e33 |
| A6.2 - Deep-sea mixed substrata |  | PA | V | AIR |  | e20 |
| A6.3 - Deep-sea sand |  | PA | V | AIR |  | e21 |
| A6.4 - Deep-sea muddy sand |  | PA | V | AIR |  | e22 |
| A6.51- Meditteranean communities of bathyal muds |  | PA | V | AIR |  | e23 |
| A6.511 - Facies of sandy muds with Thenea muricata |  | PA | V | AIR |  | e24 |
| A6.52 - Communities of abyssal muds |  | PA | V | AIR |  | e25 |
| EM_01 - Infralittoral Seabed |  | PA | V | AIR |  | e38 |
| EM_02 - Circalittoral Seabed |  | PA | V | AIR |  | e39 |
| EM_03 - Bathyal seabed |  | PA | V | AIR |  | e34 |
| EM_04 - Abyssal seabed |  | PA | V | AIR |  | e35 |
| **Special features** |  |  |  |  |  |  |
| NH - Nursery habitats | Density of nursery habitats for 16 commercial species | PR | V | AIR | MEDISEH-MAREA | e26 |
| MM - Marine mammals | Density distribution of dolphins and other marine mammals | PR | R (20 km) | Adriatic | UNEP-MAP-RAC/SPA, 2015 | e27 |
| SB - Seabirds | Distribution of seabirds in Adriatic Sea | PR | V | AIR | UNEP-MAP-RAC/SPA, 2010 | e28 |
| TU - Turtles | Distribution of turtles | PR | R (20 km) | AIR | UNEP-MAP-RAC/SPA, 2015 | e29 |
| GDR - Giant devil ray | Distribution of giant devil ray in the Adriatic | PR | R (20 km) | Adriatic | UNEP-MAP-RAC/SPA, 2015 | e32 |
| BDS - Bathypelagic component of deep sea areas* | Bathypelagic water column, characterized by mid-water  species. | PA | V | AIR | Own elaboration | e30 |

+ where PA=presence/absence, PR= probability of presence.

° acronyms of data owners or data providers as Institutions or Projects are reported in supplementary materials S3 text???.

* as suggested in Ramirez Llodra et al. 2011 for deepsea environments in general.

** source: http://www.eea.europa.eu/themes/biodiversity/eunis/eunis-habitat-classification/

§ Datasets are archived in the data repository at doi: 10.5281/zenodo.58222

**Data owners and data providers acronyms**

MEDISEH MAREA, MAREA Project MEDISEH: Mediterranean Sensitive Habitats, within the MAREA Framework “Mediterranean hAlieutic Resources Evaluation and Advice”, www.mareaproject.net/medviewer/.

**References**

UNEP-MAP-RAC/SPA. (2015). Adriatic Sea: Important areas for conservation of cetaceans, sea turtles and giant devil rays. By Holcer, D ; Fortuna, C.M and Mackelworth, P.C. Edited by Cebrian, D.,& Requena, S., RAC/SPA, Tunis ; 69 pp.

EUNIS Marine Habitat classification 2007, available at http://www.eea.europa.eu/themes/biodiversity/eunis/eunis-habitat-classification/
