# Supplementary material for: Addressing uncertainty in modelling cumulative impacts within maritime spatial planning in the Adriatic and Ionian region
Source: PLoS One. 2017 Jul 10;12(7):e0180501. doi: 10.1371/journal.pone.0180501 (PMC5503246; doi:10.1371/journal.pone.0180501)
Supplement: S4 Table — Criteria used within the expert survey to calculate the sensitivity score of environmental components to pressures deriving from maritime uses, adapted from Andersen et al. (2013). (DOCX) [file pone.0180501.s009.docx]

**S4 Table. Criteria evaluated by experts to express the EUP sensitivities**. Criteria used within the expert survey to calculate the sensitivity score of environmental components to pressures deriving from maritime uses, adapted from Andersen et al. (2013).

| **Sensitivity Criteria** | **Explanation** | **Possible answer choices** |
| --- | --- | --- |
| Pressure | Pressures generated by marine human activities, and listed in the Marine Strategy Framework Directive (MSFD), that affect the environmental components. | “No pressure” or one of the pressure listed by the MSFD, 2008 (S1 Table). |
| Pressure distance | Distance from the source at which the pressure generated by a specific activity produces effects (linear distance). | No impact, 1km, 5 km, 10 km, 20 km, > 50 km |
| Impact extent | Level at which the activity cause harm or hit the environmental component. | No impact; Individual level; Whole population; Community level |
| Impact level | Degree to which the environmental component is affected by the pressure. | No impact; Minor disturbance; Medium disturbance; devastating/Lethal |
| Recovery time | Time typically taken for the environmental component to recover after it is affected by the activity/pressure. | No impact; < 1 years; 1- 10 years; 10 – 100 years; > 100 years |
| Confidence | Level of confidence on respondent’s judgment | None; Low; Medium; High; Very High |

**References**

Andersen JH, Stock A (eds.) Mannerla M, Heinanen S, Vinther M. Human uses, pressures and impacts in the eastern North Sea. Aarhus University, DCE {Danish Centre for Environment and Energy. 136 pp. Technical Report from DCE{ Danish Centre for Environment and Energy No. 18. (2013). <http://www.dmu.dk/Pub/TR18.pdf>.

European Commission (2008), Framework Directive 2008/56/EC (MSFD) on the Strategy for the Marine Environment.
