# Supplementary material for: Addressing uncertainty in modelling cumulative impacts within maritime spatial planning in the Adriatic and Ionian region
Source: PLoS One. 2017 Jul 10;12(7):e0180501. doi: 10.1371/journal.pone.0180501 (PMC5503246; doi:10.1371/journal.pone.0180501)
Supplement: S5 Table — Locations are identified according to Walker et al. 2003. Sub-locations are identified by the modellers within the level 1 of the general uncertainty analysis. (DOCX) [file pone.0180501.s010.docx]

**S5 Table. Locations and sub-locations of uncertainty identified for the CI Assessment model.** Locations are identified according to Walker et al. 2003. Sub-locations are identified by the modellers within the level 1 of the general uncertainty analysis.

| **Location*** | **Sub-location** |
| --- | --- |
| Context | |
|  | 1.1 Geographical domain of CI analysis |
|  | 1.2 Temporal baseline of analysis of CI |
| Model |  |
|  | 2.1: oceanographic models |
|  | 2.2: spatial model of pressures on env. components |
|  | 2.3: environmental conditions as a baseline for impacts |
|  | 2.4: response of env. components to pressures |
|  | 2.5 (Stressor layers are of roughly equal importance, Uniform distribution of stressors within a pixel, Habitats either exist or are absent in a pixel, Transforming and normalizing stressors, Linear response of ecosystems to stressors, Vulnerability weights sufficiently accurate, Additive model, Linear response) |
| Input |  |
| *Human uses* | 3.1 Human uses datasets coverage |
|  | 3.2 Land-based pollution |
| *Environmental components* | 3.3 Environmental components data coverage |
|  | 3.4 Special features as marine mammals, turtles and giant devil rays |
|  | 3.5 EMODnet dataset for seabed habitats |
| *Sensitivities* | 3.6 Sensitivities |
| Parameters |  |
|  | 4.1 Grid resolution |
|  | 4.2 Number of significative pressures per each E-U relationship |
|  | 4.3 Log-normalization |
| Outcome |  |
|  | 5.1 Cumulative impacts score |

*From Walker et al. 2003

Reference

Walker W, Harremoës P, Rotmans J, Van der Sluijs JP, van Asselt MBA, Janssen P (2003) Defining Uncertainty: A Conceptual Basis for Uncertainty Management in Model-Based Decision Support. Integr Assess 4(1): 5-17.
