# Supplementary material for: Addressing uncertainty in modelling cumulative impacts within maritime spatial planning in the Adriatic and Ionian region
Source: PLoS One. 2017 Jul 10;12(7):e0180501. doi: 10.1371/journal.pone.0180501 (PMC5503246; doi:10.1371/journal.pone.0180501)
Supplement: S6 Table — The subjects who have declared the different sub-locations, levels and nature of uncertainty, are indicated as M = modelers (authors of the paper), SE = stakeholders and experts (through interviews, workshops and survey), L = literature. (DOCX) [file pone.0180501.s011.docx]

**S6 Table. Cumulative Impacts Uncertainty Matrix for the AIR.** The subjects who have declared the different sub-locations, levels and nature of uncertainty, are indicated as M=modelers (authors of the paper), SE=stakeholders and experts (through interviews, workshops and survey), L=literature.

| **Locations** | | **Level** | | | **Nature** | |
| --- | --- | --- | --- | --- | --- | --- |
|  | **Sub-Locations** | **Statistical uncertainty** | **Scenario Uncertainty** | **Recognized ignorance** | **Epistemic** | **Variability** |
| 1.Context | | |  |  |  |  |
|  | 1.1 Geographical domain of CI analysis | - | 1.1.1 Boundaries of MSP will depend on transposition in each country of the MSP directive 2014/89/EU. (M)  1.1.2 EUSAIR does not define the southern boundary of the competent area on marine domain. This is related to the undergoing MSP implementation process in relation to International and National responsibilities and to the capacity to implement transboundary MSP on high seas, which is a critical point of discussion of the MSP implementation process (M) | - | (1.1.1) | (1.1.1) |
|  | 1.2 Temporal yearly variability of analysis of CI | - | 1.2.1 The analysis is performed for year 2016, without considering annual or seasonal variations in E-U-P relationships (M). | - | (1.2.1) | (1.2.1) |
| 2. Model uncertainties | | |  |  |  | |
|  | 2.1: oceanographic models | - | 2.1.1.Dispersion of pressures and impacts not represented (Barbanti et al. 2015) (M, SE) | - | (all uncertainties entail both epistemic and variability natures, as there are some aspect that can be included with more research effort (e.g. spatial models), but there is a level of uncertainty that depends on the variability nature of the problem, basically to the capacity of improved transdisciplinary models to represent the phenomena they are molded for.) | |
|  | 2.2: spatial model of pressures on env. components | - | 2.2.1.Representativeness of the convolution (M) | - |  |  |
|  | 2.3: environmental conditions as a baseline for impacts | - | 2.3.1.Not included in the model (M, SE) | - |  |  |
| E-P | 2.4: response of env. components to pressures | - | 2.4.1. Response of the same ecosystem components in different areas is considered equal, as not necessarily all env. components answer in the same way to the same pressure in different areas (Korpinen et al. 2012); (M, SE, L) | 2.4.2. Level of resilience and identification of regime shifts (e.g. Petersen et al 2008, Graham et al 2015, Ruggeri et al 2016), (M, L) |  |  |
|  | 2.5 (Stressor layers are of roughly equal importance, Uniform distribution of stressors within a pixel, Habitats either exist or are absent in a pixel, Transforming and normalizing stressors, Linear response of ecosystems to stressors, Vulnerability weights sufficiently accurate, Additive model, Linear response of ecosystems to cumulative impacts) (Halpern and Fujita, 2013; Judd et al. 2015) (L) | | | |  |  |
| 3. Model inputs | | |  |  |  |  |
| U | 3.1. Human uses datasets coverage | 3.1.1. Missing datasets in some areas for some uses (as in Korpinen et al. 2012, and Andersen et al 2013) (M, L) | - | - | 3.1.1.1. Different monitoring effort and research effort in some segments of the case study area | 3.1.1.2. Data recollected differently by Member States and non EU Countries, as mentioned by Crise et al. 2015 |
| U | 3.2. Land based pollution | - | - | 3.2.1. No data in the model (Barbanti et al 2015) (M) | (3.2.1) |  |
| E | 3.3 Environmental components data coverage | 3.3.1. Datasets coverage: missing datasets in the Ionian for marine mammals and giant devil rays (M, L) | 3.3.2. representativeness of the species population and trends of the datasets (as for giant devil rays, Fortuna et al. 2014) (L) | 3.3.3. Response of the same ecosystem components in different areas is considered equal - Not necessarily all env. components answer in the same way to the same pressure in different areas (Korpinen et al. 2013); (M, L) | (3.3.1; 3.3.2) | (3.3.3.) |
| E | 3.4 Marine mammals, turtles and giant devil rays | - | - | 3.4.1. Datasets on sightings density but not habitat distribution models (Fortuna et al. 2014); (M, L) | (3.4.1) | - |
| E | 3.5 EMODnet dataset for seabed habitats | 3.5.1. Sensitivity analysis in Cameron, and Askew (2011), (L, E) | - | 3.5.2. Correspondence of existing Adriatic classifications (as Brambati et ali 1988) (M, L, SE) | (3.5.1, 3.5.2) | - |
| S | 3.6 Sensitivities | 3.6.1. Combination of sensitivities according to the respondents (M)  3.6.2. Spatial model computation (buffer) (M) | 3.6.3. Sensitivities scores are subjected to subjective perception of the respondents (the ones from which the experts responded) (as in Halpern et al 2008, Korpinen et al 2012) (M, L) | 3.6.4. Sensitivities that have been compiled through literature review (e.g. deepsea, A5.46; A5.47, Seabirds) relationships of pressures-impacts mechanisms are not known; (M, L, E)  3.6.5. Un-known sensitivities (the ones left without an answer from the experts) (M)  3.6.6. Sensitivities confidence represents the level of knowledge on the EUP relations | (all) | (all) representativeness and significance of sensitivities, with respect to resilience and resistance of the env. components. |
| 4. Parameter uncertainty | | |  |  |  |  |
|  | 4.1 Grid resolution | - | 4.1.1. The grid of analysis of 1km^2^ was chosen in accordance to the EEA’s reference grid in order to facilitate the use of these data within the framework of the European Union’s environmental policies (M) | - | (4.1.1) | (4.1.1) |
|  | 4.2 Number of significant pressures per each E-U relationship | - | 4.2.1. A limit of two significant pressures for each E-U combination was introduced. This parameter is meant to reduce the over-dominance of the E-U combination that have attracted more answers from the experts and were the experts did not agree (M) | - | (4.2.1) | (4.2.1) |
|  | 4.3 Log-normalization | - | 4.3.1. The log[x+1] transformation is applied to avoid an over-dominance of extreme values on the resulting cumulative human impact map and to correct typically skewed frequency distributions (M) | - | (4.3.1) | (4.3.1) |
| 5. Outcome uncertainty | | |  |  |  |  |
| CI | 5.1 Cumulative impacts score | 5.1.1 Combination of sensitivities, dispersion of the error from the input data to the results (M) | 5.1.2. Impacts dispersion considering spatial models per each E-U-P relationship (M, L) | 5.1.3. Mechanisms of combination of impacts (synergetic, multiplicative or reducing effects, from Halpern et al 2008) (M, L)  5.1.4. Ecological significance of the potential impacts on environmental components, considering not only high CI but low signals on population or community levels in long-term perspective (M, E) | (5.1.1, 5.1.2.) | (5.1.2, 5.1.3, 5.1.4) |

**References**

Barbanti A, Campostrini P, Musco F, Sarretta A, Gissi E (eds.) Developing a Maritime Spatial Plan for the Adriatic-Ionian Region. CNR-ISMAR, Venice, IT; 2015.

Andersen JH, Stock A (eds.) Mannerla M, Heinanen S, Vinther M. (2013), Human uses,pressures and impacts in the eastern North Sea. Aarhus University, DCE Danish Centre for Environment and Energy. 136 pp. Technical Report from DCE { Danish Centre for Environment and Energy No. 18http://www.dmu.dk/Pub/TR18.pdf.

Brambati A, Ciabatti M, Fanzutti GP, Marabini F, Marocco R (1988a) Carta sedimentologica dell ~ OAdriatico settentrionale, 1: 250000. Consiglio nazionale delle ricerche.

Cameron A, Askew N. EUSeaMap-Preparatory Action for development and assessment of a European broad-scale seabed habitat map final report. Cameron, A., Askew, N.(Eds.); 2011, 240.

Crise A, Kaberi H, Ruiz J, Zatsepin A, Arashkevich E, Giani M et al. (2015) A MSFD complementary approach for the assessment of pressures, knowledge and data gaps in Southern European Seas: The PERSEUS experience. Mar Pollu Bull 95(1): 28-39.

Fortuna CM, Kell L, Holcer D, Canese S, Filidei Jr E, Mackelworth P, Donovan G (2014) Summer distribution and abundance of the giant devil ray (Mobula mobular) in the Adriatic Sea: Baseline data for an iterative management framework. Scientia Marina 78(2): 227-237.

Graham NA, Jennings S, MacNeil MA, Mouillot D, Wilson SK (2015). Predicting climate-driven regime shifts versus rebound potential in coral reefs. Nature, 518(7537), 94-97

Halpern BS, Fujita R (2013) Assumptions challenges, and future directions in cumulative impact analysis. Ecosphere 4(10): art131.

Halpern BS, McLeod KL, Rosenberg AA, Crowder LB (2008). Managing for cumulative impacts in ecosystem-based management through ocean zoning. Ocean & Coastal Management, 51(3), 203-211.

Judd A, Backhaus T, Goodsir F (2015) An effective set of principles for practical implementation of marine cumulative effects assessment. Env Sci& Pol, 54:254-262.

Korpinen S, Meidinger M, Laamanen M (2013) Cumulative impacts on seabed habitats: An indicator for assessments of good environmental status. Mar Pollut Bull 74(1): 311-319.

Petersen JK, Hansen JW, Laursen MB, Clausen P, Carstensen J, Conley DJ (2008). Regime shift in a coastal marine ecosystem. Ecological Applications, 18(2), 497-510.

Ruggeri P, Splendiani A, Occhipinti G, Fioravanti T, Santojanni A, Leonori I, et al. (2016). Biocomplexity in Populations of European Anchovy in the Adriatic Sea. PloS one, 11(4), e0153061.
