# Supplementary material for: Addressing uncertainty in modelling cumulative impacts within maritime spatial planning in the Adriatic and Ionian region
Source: PLoS One. 2017 Jul 10;12(7):e0180501. doi: 10.1371/journal.pone.0180501 (PMC5503246; doi:10.1371/journal.pone.0180501)
Supplement: S7 Table — (DOCX) [file pone.0180501.s012.docx]

**S7 Table. Data availability per human uses U for different geographical areas.**

|  | Human uses | | | | | | | | | | | | | | | |
| --- | --- | --- | --- | --- | --- | --- | --- | --- | --- | --- | --- | --- | --- | --- | --- | --- |
| Geographical areas (terms) | Aquaculture | Cables and Pipelines | Coastal and Maritime Tourism | Coastal Defence Work | Dumping area for dredging | LNGs | Maritime Transport | Military areas | Naval Base Activities | Off-shore sand deposit | Oil and Gas Extraction | Oil and Gas Research | Renewable Energy facilities | Small scale Fishery | Trawling | Tot |
| AIR |  | 1 |  |  |  | 1 |  |  | 1 |  |  | 1 | 1 |  | 1 | 6 |
| Adriatic |  | 1 |  |  |  | 1 | 1 | 1 | 1 |  |  | 1 | 1 |  | 1 | 8 |
| Albania |  | 1 |  |  |  | 1 | 1 | 1 | 1 |  |  | 1 | 1 |  | 1 | 8 |
| Italian Adriatic |  | 1 |  |  |  | 1 | 1 | 1 | 1 | 1 | 1 | 1 | 1 | 1 | 1 |  |
| Abruzzo | 1 | 1 | 1 | 1 |  | 1 | 1 | 1 | 1 | 1 | 1 | 1 | 1 | 1 | 1 | 14 |
| Apulia |  | 1 | 1 | 1 |  | 1 | 1 | 1 | 1 |  |  | 1 | 1 |  | 1 |  |
| Adriatic Apulia | 1 | 1 |  | 1 |  | 1 | 1 | 1 | 1 | 1 | 1 | 1 | 1 | 1 | 1 | 13 |
| Emilia Romagna | 1 | 1 | 1 | 1 | 1 | 1 | 1 | 1 | 1 | 1 | 1 | 1 | 1 | 1 | 1 | 15 |
| Friuli Venezia Giulia |  | 1 |  |  |  | 1 | 1 | 1 | 1 | 1 | 1 | 1 | 1 | 1 | 1 | 11 |
| Marche | 1 | 1 | 1 | 1 |  | 1 | 1 | 1 | 1 | 1 | 1 | 1 | 1 | 1 | 1 | 14 |
| Molise | 1 | 1 | 1 | 1 |  | 1 | 1 | 1 | 1 | 1 | 1 | 1 | 1 | 1 | 1 | 14 |
| Veneto | 1 | 1 | 1 | 1 |  | 1 | 1 | 1 | 1 | 1 | 1 | 1 | 1 | 1 | 1 | 14 |
| Croatia | 1 | 1 |  |  |  | 1 | 1 | 1 | 1 |  | 1 | 1 | 1 | 1 | 1 | 11 |
| Montenegro | 1 | 1 |  |  |  | 1 | 1 | 1 | 1 |  |  | 1 | 1 |  | 1 | 9 |
| Slovenia | 1 | 1 | 1 |  |  | 1 | 1 | 1 | 1 |  |  | 1 | 1 | 1 | 1 | 11 |
| Ionian |  | 1 |  |  |  | 1 |  |  | 1 |  |  | 1 | 1 | 1 | 1 | 7 |
| Italian Ionian |  | 1 |  |  |  | 1 |  | 1 | 1 |  |  | 1 | 1 | 1 | 1 | 8 |
| Basilicata |  | 1 |  |  |  | 1 |  | 1 | 1 |  |  | 1 | 1 | 1 | 1 | 8 |
| Calabria |  | 1 |  |  |  | 1 |  | 1 | 1 |  |  | 1 | 1 | 1 | 1 | 8 |
| Sicilia |  | 1 |  |  |  | 1 |  | 1 | 1 |  |  | 1 | 1 | 1 | 1 | 8 |
| Ionian Apulia |  | 1 | 1 | 1 |  | 1 | 1 | 1 | 1 |  |  | 1 | 1 |  | 1 | 10 |
| Greece | 1 | 1 | 1 |  |  | 1 |  |  | 1 |  |  | 1 | 1 | 1 | 1 | 9 |
