# Supplementary material for: Addressing uncertainty in modelling cumulative impacts within maritime spatial planning in the Adriatic and Ionian region
Source: PLoS One. 2017 Jul 10;12(7):e0180501. doi: 10.1371/journal.pone.0180501 (PMC5503246; doi:10.1371/journal.pone.0180501)
Supplement: S1 File — A vocabulary of geographical areas was composed to characterize the geographical scope of each dataset. It consists of 22 terms, which can be modified and new terms can be added. Each term was associated to a specific geometry and related boundary, which was input in the model to calculate the data availability index (DAI). (DOCX) [file pone.0180501.s013.docx]

### S1 File. Gazetteer of spatial coverage of input dataset. A vocabulary of geographical areas was composed to characterize the geographical scope of each dataset. It consists of 22 terms, which can be modified and new terms can be added. Each term was associated to a specific geometry and related boundary, which was input in the model to calculate the data availability index (DAI).

**Terms**

1. Abruzzo: Italian territorial waters and high seas in front of Abruzzo Region till the median line;
2. Adriatic Apulia: Italian territorial waters and high seas in front of Apulia Region in the Adriatic Sea (excluding Gulf of Taranto) till the median line;
3. Adriatic: Adriatic Sea, according to EUNIS EMODnet (2014);
4. Adriatic Italy: Italian territorial waters and high seas of Adriatic Sea till the median line;
5. AIR: Adriatic Ionian Region, on the geographical domain of the study;
6. Albania: Albanian territorial waters and high seas till the median line with Italy;
7. Apulia: Italian territorial waters and high seas in front of Apulia Region till the median line;
8. Basilicata: Italian territorial waters and high seas in front of Basilicata Region in the Ionian Sea;
9. Calabria: Italian territorial waters and high seas in front of Calabria Region in the Ionian till the median line with Greece;
10. Croatia: Croatian territorial waters and the area of Fishery and Ecologically protected zones of Croatia till the median line with Italy (the boundary with Slovenia is currently under dispute through international arbitration);
11. Emilia Romagna: Italian territorial waters and high seas in front of Emilia Romagna Region till the median line;
12. Friuli Venezia Giulia: Italian territorial waters and high seas in front of Friuli Venezia Giulia till the median line;
13. Greece: Greek territorial waters and high seas on continental shelf till the median line with Italy
14. Ionian: Ionian Sea included in the case study area (AIR);
15. Italian Adriatic: Italian territorial waters and high seas till the median line with Croatia, Slovenia, Montenegro and Albania;
16. Italian Ionian: Italian territorial waters and high seas on continental shelf till the median line with Greece in the Ionian, considering the area included in the case study (AIR);
17. Marche: Italian territorial waters and high seas in front of Marche Region till the median line;
18. Molise: Italian territorial waters and high seas in front of Molise till the median line;
19. Montenegro: territorial waters and high seas till the median line with Italy;
20. Sicilia: Italian territorial waters and high seas in front of Sicilia Region in the Ionian Sea till the median line with Greece;
21. Slovenia: Slovenian waters in the Adriatic Sea (the boundary with Croatia is currently under dispute through international arbitration);
22. Veneto: Italian territorial waters and high seas in front of Veneto Region till the median line.

**References**

EMODnet Secretariat. EMODnet Thematic Lot n° 3, EMODnet Phase 2 – Annual (interim) report, Reporting Period: Sept. 2013 to Aug. 2014. 09/09/2014, [accessed on 31/05/2016] http://www.emodnet-seabedhabitats.eu/default. aspx?page=2024
